# CREB controls cortical circuit plasticity and functional recovery after stroke

**DOI:** 10.1038/s41467-018-04445-9

**Published:** 2018-06-08

**Authors:** L. Caracciolo, M. Marosi, J. Mazzitelli, S. Latifi, Y. Sano, L. Galvan, R. Kawaguchi, S. Holley, M. S. Levine, G. Coppola, C. Portera-Cailliau, A. J. Silva, S. T. Carmichael

**Affiliations:** 1 Department of Neurology, David Geffen School of Medicine, University of California Los Angeles, Los Angeles, CA 90095 USA; 2 Department of Neurobiology, David Geffen School of Medicine, University of California Los Angeles, Los Angeles, CA 90095 USA; 3 Department of Psychology, David Geffen School of Medicine, University of California Los Angeles, Los Angeles, CA 90095 USA; 4 Department of Psychiatry and Biobehavioral Sciences, David Geffen School of Medicine, University of California Los Angeles, Los Angeles, CA 90095 USA; 5 Integrative Center for Learning and Memory, David Geffen School of Medicine, University of California Los Angeles, Los Angeles, CA 90095 USA; 6 Brain Research Institute, David Geffen School of Medicine, University of California Los Angeles, Los Angeles, CA 90095 USA; 7 Program in Neurogenetics, Department of Neurology, David Geffen School of Medicine, University of California Los Angeles, Los Angeles, CA 90095 USA; 8 Department of Psychiatry, Semel Institute for Neuroscience and Human Behavior, David Geffen School of Medicine, University of California Los Angeles, Los Angeles, CA 90095 USA

## Abstract

Treatments that stimulate neuronal excitability enhance motor performance after stroke. cAMP-response-element binding protein (CREB) is a transcription factor that plays a key role in neuronal excitability. Increasing the levels of CREB with a viral vector in a small pool of motor neurons enhances motor recovery after stroke, while blocking CREB signaling prevents stroke recovery. Silencing CREB-transfected neurons in the peri-infarct region with the hM4Di-DREADD blocks motor recovery. Reversing this inhibition allows recovery to continue, demonstrating that by manipulating the activity of CREB-transfected neurons it is possible to turn off and on stroke recovery. CREB transfection enhances remapping of injured somatosensory and motor circuits, and induces the formation of new connections within these circuits. CREB is a central molecular node in the circuit responses after stroke that lead to recovery from motor deficits.

## Abstract (web summary)

Increasing excitability in the peri-infarct area enhances motor recovery after stroke. Here the authors show that expressing CREB, a transcription factor known for its role in synaptic plasticity, or increasing activity of CREB-expressing cells near the stroke site improves recovery in an effect that is strong enough that it can be used to turn on and off motor recovery after stroke.

## Introduction

Stroke is the leading cause of adult disability because of the brain’s limited capacity to repair^1^. Approaches that increase neuronal excitability, such as anodal direct current stimulation or transcranial magnetic stimulation, enhance motor performance after stroke^2–6^. In rodent models of stroke, pharmacogenetic treatments that enhance neuronal excitability in peri-infarct cortex adjacent to the stroke also promote motor recovery^7,8^. These data in humans and rodent models of stroke support the concept that enhancing neuronal excitability in motor circuits ipsilateral to the stroke may increase the function in these partially damaged areas and therefore promote recovery.

The transcription factor cAMP-response-element binding protein (CREB) enhances long-term synaptic plasticity and increases neuronal excitability^9–12^. Viral CREB transduction in neurons boosts baseline firing rate and the formation of long-term potentiation (LTP)^10–12^. CREB also plays a role in cortical remapping to environmental alterations^13,14^. In these functions, CREB increases spine density in neurons^5,15^, altering local neuronal connectivity. Interestingly, stroke recovery is associated with dramatic spine plasticity in the peri-infarct cortex, with an increase in spine density over baseline values in some regions^6^. These data indicate that CREB-dependent transcription has a critical role in the modulation of neuronal excitability and in long-lasting alterations in circuit structure during cortical plasticity and memory. We hypothesized that CREB function in a localized network of motor cortical neurons near the stroke site might enhance motor recovery by facilitating remapping of local cortical networks.

## Results

### CREB in a pool of neurons induces recovery after stroke

**Fig. 1:**
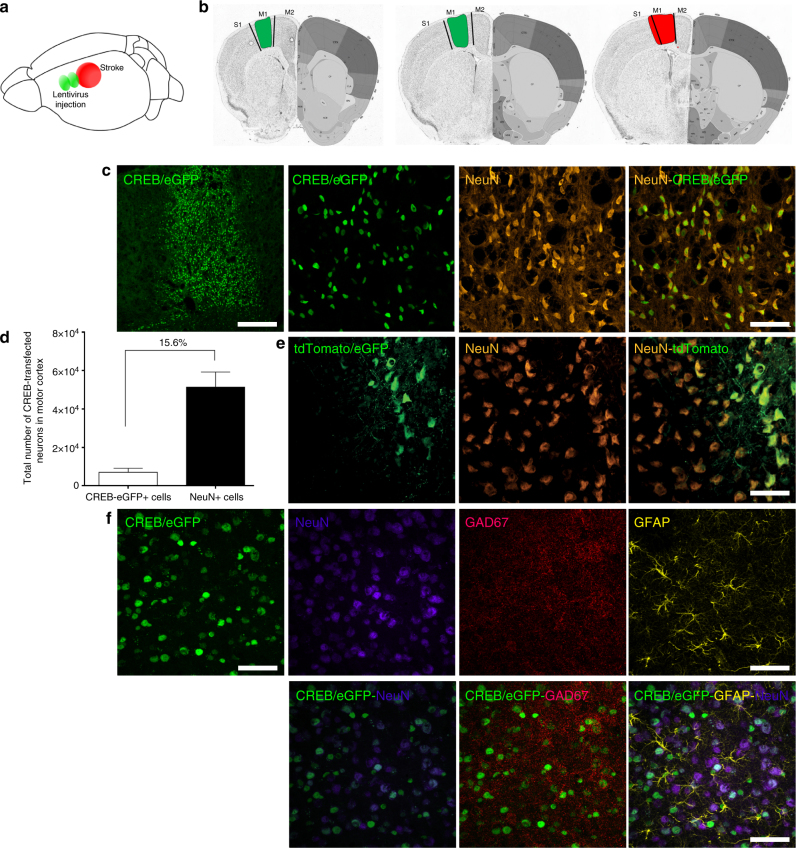
Lentiviral CREB expression in cortical pyramidal neurons. **a** Schematic shows location of stroke area (red) and two lentivirus injections (green) in the peri-infarct area. **b** Atlas-based^22^ schematic of location of lentivirus injection (green) and stroke (red). **c** Left: Lenti-CREB-eGFP in peri-infarct cortex at the time of stroke, 7 days after injection and after stroke induction. Transfected cells form a column in cortex. Top is the pial surface, bottom is the white matter. Scale bar = 300 μm. Right panels: CREB-eGFP staining (green, infected cell) in peri-infarct tissue, co-localize with NeuN staining (orange) 4 weeks after stroke. Scale bar = 50 μm. **d** Stereological quantification of motor cortex CREB-induced cells (CREB-eGFP+ cells) relative to the whole motor cortex neuronal pool (NeuN+ cell ± SEM). *n* = 4 (eight brain sections for each mouse). **e** Immunohistochemical staining in peri-infarct M1 in mice transfected with control virus (green, tdtomato-eGFP) overlapping with NeuN staining (orange) 4 weeks after stroke. Scale bar = 50 μm. **f** Left: immunohistochemical staining in peri-infarct M1 in mice transfected with Lenti-Creb-eGFP (green) overlapping with NeuN staining (purple); Middle: immunohistochemical staining showing inhibitory neurons (GAD67, glutamate decarboxylase 67); Right: immunohistochemical staining showing that there are very rare Lenti-CREB infected cells that double stain for GAD67, 4 weeks after stroke. Scale bar = 100 μm. M1 primary motor cortex, M2 secondary motor cortex, S1 primary somato sensory cortex

To determine the role of CREB in motor recovery after stroke, we increased its expression in a small pool of neurons in motor and premotor cortex anterior to the site of the stroke (Fig. 1a, b; Supplementary Fig. 1). A photothrombotic stroke was produced in the forelimb motor cortex. This model produces long-lasting behavioral deficits in motor function^8,16–20^ in which motor and premotor sites play a causal role in motor recovery^17–20^. In this lentiviral gene delivery, CREB-eGFP expression is under the regulation of the excitatory neuron-specific αCaMKII promoter along with enhanced green fluorescent protein (CaMKIIa_HA/AlstR_CREB/eGFP, referred as lenti-CREB; Fig. 1c). Control virus was eGFP/tdTomato (CamkIIa_HA/AlstR_eGFP/tdTomato; Fig. 1e). Unlike adeno-associated virus (AAV), lentivirus produces a restricted column of transfected cortical neurons (Fig. 1c). Stereological quantification shows that lentiviral CREB expression is present in 8884 ± 2753 neurons in the motor cortex (Fig. 1d). Consistent with the specificity of the αCaMKII promoter, there was no co-localization of lentivirus-CREB with markers of inhibitory neurons (glutamate decarboxylase 67; GAD67) or astrocytes (glial fibrillary acidic protein; GFAP) (Fig. 1f; Supplementary Figs. 2–4). Thus, this approach increases the expression of wild-type CREB, delivering a gain of function in a tightly circumscribed region of motor cortex adjacent to the stroke site, corresponding to roughly 16% of all neurons in motor cortex (total neuronal nuclei (NeuN) positive neurons: 57,000 ± 7900; Fig. 1d). This lentivirus-CREB approach increases neuronal excitability (Supplementary Fig. 5).

**Fig. 2:**
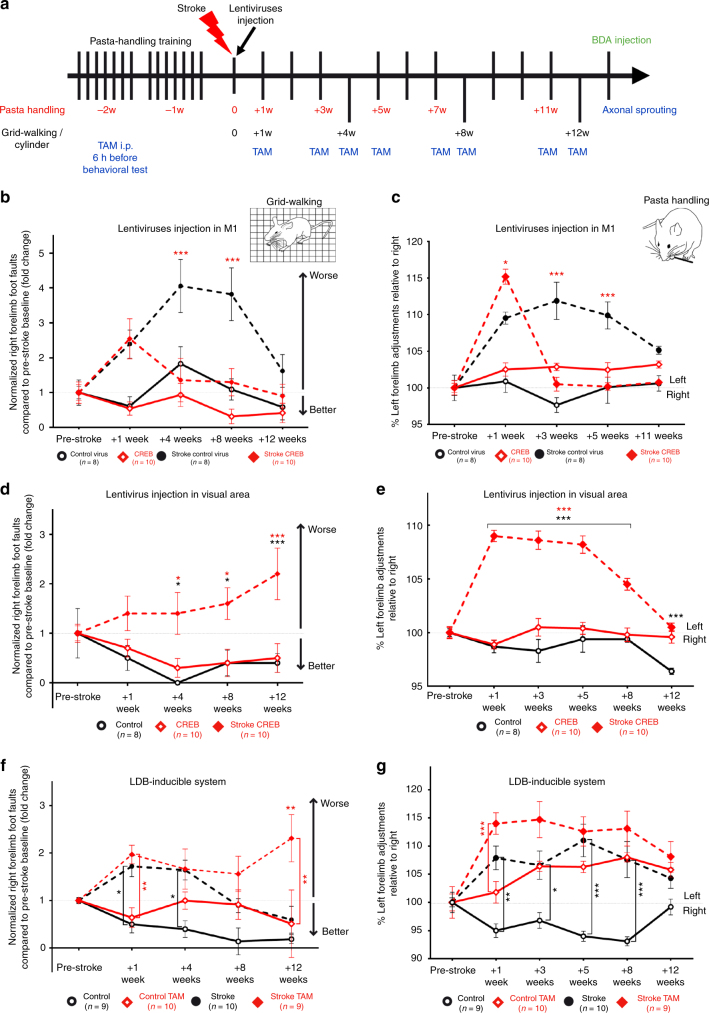
Lentiviral CREB in peri-infarct motor enhances motor recovery. **a** Experimental timeline of behavioral studies. TAM taxomifen. **b**, **d**, **f** Gridwalking tasks of forelimb function in gait. *Y* axis is percentage of footfaults of the right (affected) forelimb contralateral to the stroke. **c**, **e**, **g** Pasta handling task of distal forelimb function. *Y* axis is the percentage of left forelimb adjustments (unaffected forepaw) relative to right forepaw (affected forepaw). **b**, **c** CREB gain of function. Stroke + CREB induction in motor cortex produced a significant recovery in forelimb function compared with stroke + Control virus (***p < 0.005) over 3–4 weeks after stroke, respectively in pasta handling adjustments and gridwalking. **d, e** Stroke + CREB induction in posterior parietal cortex did not produce recovery in forelimb function either in gridwalking or pasta handling over 8 weeks after stroke. **f, g** CREB loss of function. Activating CREB^IR^ with TAM 6 h before behavioral testing (stroke + TAM) reduces motor control and blocks the pattern of motor recovery seen in stroke alone (Stroke + Saline). *P *<* 0.05, **P < 0.01, ***P <  0.005. Error bars are SEM. Statistics are two way ANOVA followed by Bonferroni post hoc test. *n* = 8 for control virus and stroke control virus; *n* = 10 for CREB and stroke CREB

Lentivirus was injected immediately after cortical stroke, expressing detectable CREB levels approximately 7 days after the stroke (Fig. 1c; Supplementary Figs. 1–4). Behavioral performance in motor control was measured over 12 weeks in grid-walking, pasta-handling (capellini test), and cylinder tests (Fig. 2a; Supplementary Figs. 6–9). These tasks measure the pattern of movement of the limbs of animals during locomotion, skilled forelimb use, and in exploratory forelimb use, respectively^20–22^. Stroke impairs motor control in all three tasks (Fig. 2a; Supplementary Figs. 5–9). In stroke control virus, recovery was observed only by 12 weeks in grid walking (Stroke control virus vs Stroke CREB; 4 weeks: ***P < 0.001; 8 weeks: **P < 0.005; *F* (3, 160) = 19.90), while there was still a persistent deficit in the pasta-handling task (Stroke control virus vs Stroke CREB; 3 weeks: ****P < 0.0001, 5 weeks: ****P < 0.0001, *F* (3, 160) = 36.26; Fig. 2b, c). Moreover, we observed that mice with stroke continued to use the non-affected forelimb (left paw) in the cylinder task over 12 weeks after stroke (*P* = 0.0008, *F* (3, 170) = 5.799; Supplementary Fig. 6). Remarkably, lenti-CREB delivery to a pool of motor cortical neurons anterior to the stroke site significantly improves functional recovery of motor control within 3–4 weeks in grid and pasta handling tasks (grid-walking: 4 weeks ***P < 0.001, *F* (3, 160) = 19.90; pasta handling: 3 weeks ****P < 0.0001, *F* (3, 160) = 36.26; Fig. 2b, c). An improvement in forelimb use with lenti-CREB also occurs in the cylinder task even though not statistically significant (Supplementary Fig. 6).

To further evaluate the role of CREB in stroke recovery, this experiment was repeated in a larger stroke model, which involves the striatum, subcortical white matter, and cortex (Supplementary Fig. 11a, b). Viral delivery and behavioral testing were as for the cortical stroke model, except only gridwalking and pasta handling were tested because of the variability of the cylinder task. The behavioral deficits are worse in this larger stroke, and there is little recovery after months from the infarct in both gridwalking and pasta handling (Supplementary Fig. 11c, d). Beginning at 7 weeks in pasta handling (*F* (2, 111) = 22.44; *P* < 0.0012) and at 11 weeks in gridwalking (*F* (2, 124) = 41.63; *P* < 0.0093), there is a significant improvement in motor performance in lenti-CREB compared to stroke + control virus. In all stroke behavioral studies, cortical and subcortical stroke and in the ligand binding domain (LBD) CREB loss of function stroke model (below), there is no difference in stroke size across CREB and control conditions (Supplementary Fig. 11e–h). These studies show that CREB induction in a small pool of excitatory motor cortical neurons is sufficient to accelerate functional motor recovery of the contralateral forelimb after stroke in two different stroke models.

CREB-induced recovery is specific to the neuronal circuits with CREB-transfected cells in motor cortex anterior to the stroke, as lenti-CREB injection into cortex at a corresponding site immediately posterior to the stroke lesion (posterior parietal association area, PTLp^22^) does not enhance motor recovery after stroke, indicating the selective nature of CREB function in peri-infarct motor cortical neurons (Fig. 2d, e; Supplementary Fig. 7).

Motor recovery in this and other rodent stroke models occurs spontaneously. During behavioral testing, such as walking on the challenging grid, mice make initial mistakes in the testing time epoch and then improve their performance (Supplementary Fig. 9). To determine if CREB plays a role in this process of motor learning during spontaneous recovery after stroke, we used the LBD-inducible system to temporally and reversibly repress CREB function using Tamoxifen (TAM)^23^, TAM delivery disrupts memory consolidation in contextual fear conditioning tasks with a tight temporal window 6–12 h before the conditioning stimulus^23^. LBD-CREB mice were administered TAM or vehicle (saline) 6 h before behavioral testing. Blockade of CREB signaling immediately prior to motor testing in mice prevented the time-dependent improvement in performance in grid-walking and reduced the overall level of motor performance. (Fig. 2f; Supplementary Fig. 8). Stroke in LBD-CREB mice in which TAM is administered prior to testing shows that motor recovery does not improve over the course of the post-stroke period, up to 2 months (Fig. 2f). Inhibiting CREB in the LBD-CREB mice immediately before motor testing reduces the motor performance in gait testing, such that LBD-CREB mice given TAM prior to testing show a flat recovery curve and persistent motor control deficit in grid walking (Fig. 2f; Supplementary Fig. 9). CREB inhibition produced a trend toward worse motor performance in pasta-handling (Fig. 2g; Supplementary Fig. 8). Overall, these data indicate that CREB gain of function improves motor recovery after stroke, that this is specific to motor cortical circuits adjacent to the stroke site, and that CREB loss of function inhibits motor control after stroke.

### Switching on or off recovery of motor function after stroke

**Fig. 3:**
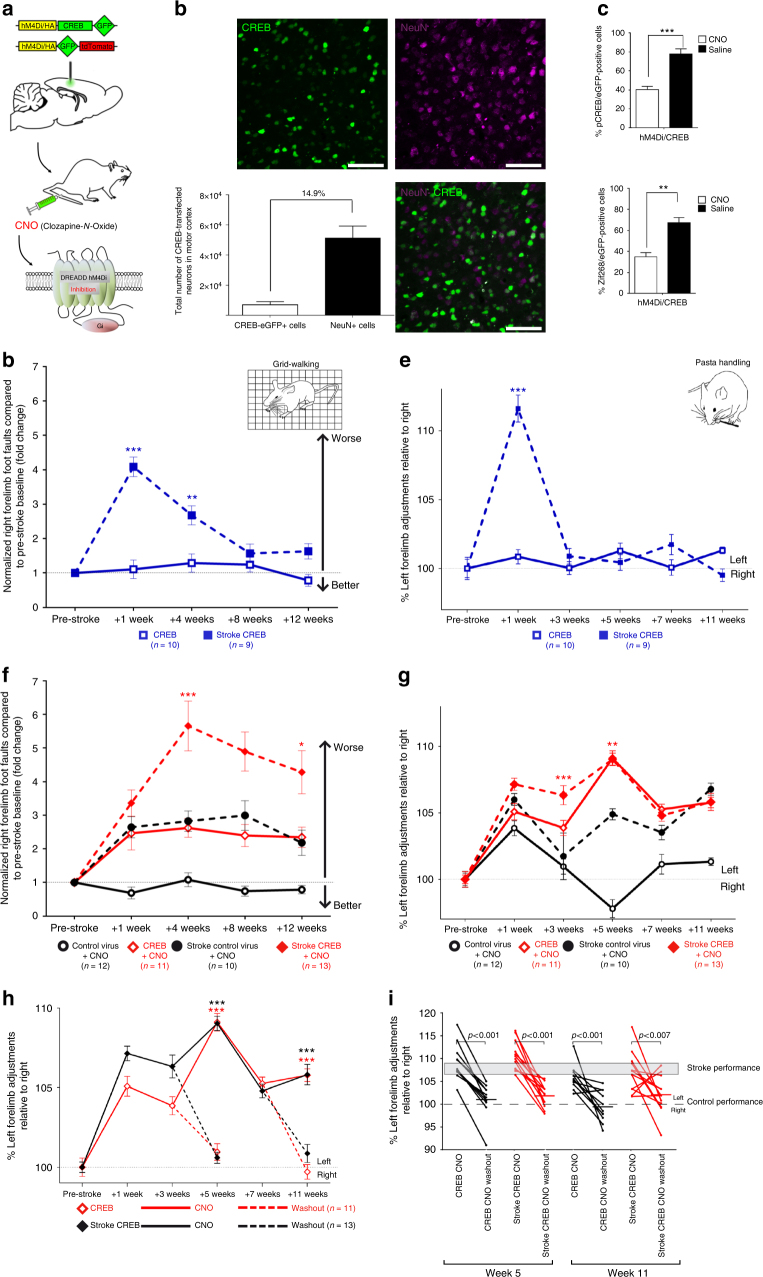
hM4Di/CREB inhibition of cortical neurons after stroke blocks motor recovery. **a** Schematic shows the process by which DREADD hM4Di-CREB when activated by CNO (30 min before behavioral tasks) can lead to selective inactivation of neurons in motor cortex in which CREB is induced. **b** Immunohistochemical staining in peri-infarct M1 in mice transfected with hM4Di-CREB/eGFP (green, infected cells) overlapping with NeuN staining (purple) 4 weeks after stroke. Scale bar = 50 μm. Stereological quantification of motor cortex M1 CREB-induced cells (CREB-eGFP+ cells) relative to the whole motor cortex (NeuN+ cells). **c** Stereological quantification of two immediate early genes, pCREB (phospho-CREB) and Zif268, in hM4Di/CREB-induced neurons in M1 after CNO or Saline administration during pasta handling motor task. ** *P* < 0.05,***P *<* 0.005, one way ANOVA followed by Bonferroni post hoc test *n* = 4. Eight brain sections for each mouse. **d**,** f** Gridwalking tasks of forelimb function in gait. *Y* axis is percentage of the number of footfaults of the right (affected) forelimb contralateral to the stroke. **e**, **g** Pasta handling tasks. *Y* axis is the percentage of left forelimb adjustments (unaffected forepaw) relative to right forepaw (stroke-affected forepaw). **d**, **e** CREB gain of function. In the absence of CNO, Stroke + hM4Di-CREB induction in peri-infarct M1 produces a significant recovery in forelimb function over 3–4 weeks after stroke, respectively in pasta handling adjustments and gridwalking, as with studies in Fig. 2b, d. *n* = 13 for stroke CREB CNO, *n* = 10 for CREB, and *n* = 9 for stroke CREB. **f**, **g** Selective inactivation with hM4Di-CREB of neurons in motor cortex (CNO administration) blocks motor recovery compared to mice with stroke + control virus either in grid walking or in pasta handling adjustments (**P < 0.05, ***P < 0.005, two way ANOVA followed by Bonferroni post hoc test). **h**, **i** Pasta handling graphs show motor recovery induced by hM4Di-CREB induction in the same mice in the absence of CNO (washout, mice tested day after CNO administration) at 5 and 11 weeks after stroke. *n* = 12 for control virus CNO, *n* = 11 for CREB CNO, and *n* = 10 for stroke control virus CNO (**h**). **i** Individual animals in pasta handling performance after CNO administration and after washout and 5 and 11 weeks after stroke. Gray bar shows the average performance of stroke-only mice, taken from data in Fig. 1c–e

To directly test the role of CREB-expressing neurons in motor recovery, we selectively inactivated the neurons in motor cortex with lentivirus expressed-CREB. Viruses were transfected into motor cortex anterior to the stroke as in the previous experiments. In these experiments, however, we included the hM4Di DREADD receptor to inducibly inactivate these neurons^24^ (hM4Di/CREB, Fig. 3a, b). We also included several controls. First, to control for the CREB effect, a cohort of mice received the same viral construct with only hM4Di (no CREB: referred to as hM4Di, Supplementary Fig. 9). Also, cohorts of mice received hM4Di/CREB but did not receive the hM4Di ligand (Clozapine-*N*-Oxide, CNO) in both stroke and control groups, to control for the effect of CNO. Stereological quantification shows that these viruses transfect 7100 ± 2070 neurons in motor cortex (Fig. 3b), similar to the original lenti-CREB constructs (Supplementary Fig. 3).

Mice received injections of lenti-CREB + hM4Di or hM4Di alone at the time of stroke. As with the previous experiments, these viruses express protein beginning at one week after injection so that the effect is tested during stroke recovery and not in the acute phase of stroke cell death. CNO administration to mice with hM4Di/CREB reduces the immediate early genes Zif268 and phospho-Creb (pCREB) as measured stereologcially, co-localized with viral transfection, indicating a significant reduction in neuronal activation in cells in which hM4Di is expressed (**P < 0.005 and ***P < 0.001, respectively, Student’s *t*-test; Fig. 3c). Mice were tested behaviorally over 12 weeks after stroke (Fig. 3d–i). Saline or CNO was delivered 30 min before each behavioral test. This strategy selectively inactivates neurons in motor cortex just prior to behavioral testing.

As shown above, with the CREB lentivirus (saline administration), overexpression of CREB with the hM4Di/CREB vector (but no CNO) enhances motor recovery in stroke. hM4Di/CREB saline mice demonstrate greater control and faster speed in eating pasta, a reduction in the number of foot faults in grid-walking, improved gait, and preference for the right (affected) paw in exploratory forelimb use in cylinder task starting at 4 weeks after stroke (****P < 0.0001, *F* (5, 300) = 98.15; Fig. 3d,e, Supplementary Fig. 9). These results replicate the earlier findings that lentivirus induction of CREB enhances motor recovery (Fig. 2b, c).

Importantly, inactivating CREB-transfected cells with CNO blocks the improved motor control in recovery after stroke. Mice with hM4Di/CREB + CNO-stroke perform significantly worse in gait, skilled pasta handling and affected forelimb use in rearing than saline controls (****P < 0.0001, *F* (5, 372) = 63.86; Fig. 3f, g, Supplementary Fig. 9). The poor motor control is particularly noticeable in the grid walking task, in which hM4Di/CREB mice when given CNO prior to testing commit roughly twice as many foot-faults compared to stroke-alone or stroke + hM4Di + CNO (no CREB) (Fig. 3f). In the pasta handling task, both groups of mice in which CREB is induced (with stroke and in non-stroke) perform worse than stroke + hM4Di + CNO (****P < 0.0001, *F* (5, 372) = 63.86; Fig. 3g, Supplementary Fig. 10), indicating that inhibition of CREB transduced neurons causes a worse motor deficit than inhibition of a similar number of neurons without CREB induction. These data indicate that acute inactivation of CREB-transfected motor cortical neurons after stroke does not just block the normal recovery of motor performance after stroke, but causes a marked deterioration of limb control that is much greater than that produced by stroke-alone. The results suggest that CREB-expressing neurons are preferentially incorporated into stroke recovering circuits, a result consistent with studies that demonstrated that CREB-expressing neurons are preferentially incorporated into memory engrams^24–26^.

Accordingly, inactivation of CREB-expressing neurons in the non-stroke groups caused a significant deficit in motor control in pasta handling and grid-walking (Fig. 2b) compared with mice transfected with viral vectors with hM4Di but without CREB (****P < 0.0001, *F* (5, 372) = 63.86; ****P < 0.0001, *F* (5, 372) = 63.86; Fig. 3f, g, Supplementary Fig. 10). The degree of motor deficit triggered by inactivation of CREB-expressing neurons in the normal brain is similar to that observed after stroke. Again, this impairment is not seen after inactivation of cells transfected with viral vectors that do not include CREB (Fig. 3f, g). Also, DREAD-induced inactivation of motor cortical neurons alone does not impair motor control (Supplementary Fig. 12), indicating that it is first the induction of CREB, inducing circuit plasticity, and then the acute inactivation of CREB-induced neurons that produces deficits in motor control (non-stroke) or the recovered motor function (stroke).

Altogether, these experiments indicate that inactivating a pool of excitatory motor cortical neurons (15% of the motor cortex) with hM4Di has no observable effect on motor performance (Fig. 3b; Supplementary Fig. 12). However, inactivating this population of neurons when CREB is first induced in them profoundly impairs motor performance in both control (non-stroke) and stroke mice, a result consistent with a role for CREB in neuronal allocation of motor recovery. As a final test of the role of CREB-transfected motor neurons in recovery, we performed a washout study (Fig. 3h, i). Mice were first tested with CNO induction and then tested again the next day, after CNO had cleared. If CREB-transfected motor neurons are indeed causally mediating recovery of motor performance, then CNO administration should eliminate recovery of motor performance (see above), and this recovery should re-emerge after CNO wash out. This is indeed the pattern. Testing on weeks 5 and 11 in the same animals in back to back days, first with CNO administered and then with washout, shows that the recovered motor performance that is induced by CREB is present without CNO, and is blocked with CNO in individual animals (Fig. 3h, i). This experiment means that motor recovery after stroke can literally be turned on or turned off in the same animals.

### CREB induction alters movement maps in motor cortex

**Fig. 4:**
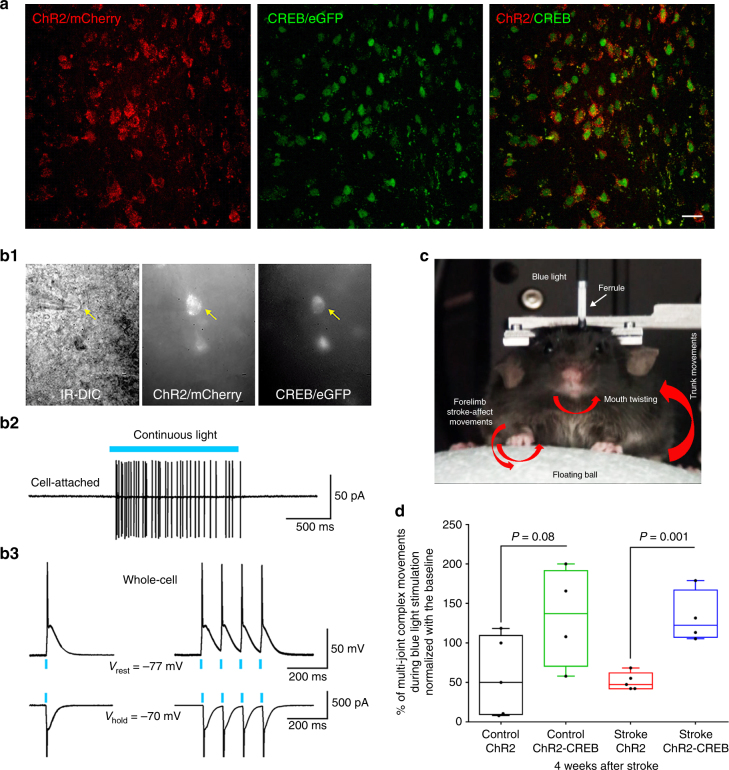
Lentiviral CREB expression induces greater multi-joint movements after stroke. **a** Immunohistochemical staining of pyramidal cells (CAMKIIα promoter drives expression in pyramidal neurons) in peri-infarct M1 in mice transfected with Lentiviral ChR2-mCherry (left) and lentiviral CREB-eGFP (middle). Right: Co-localization of ChR2 and CREB lentiviruses 4 weeks after stroke, showing nearly complete co-transfection with these two viruses. Scale bar = 50 μm. **b** Whole cell recording shows a pyramidal cell (cortex) transfected with lenti-ChR2/mCherry. The cell displayed action potentials (AP) in cell attached mode with blue light (4 mW) and tracked higher frequency light stimulation (10 Hz). At −70 mV, blue light (4 mW, 0.5 ms pulse) triggers an inward current as expected. **c** Setup of head-fixed mouse allowed to run freely on a floating Styrofoam ball to detect multi-joint movements during brief pulses of blue light stimulation (pulse length 5 ms, pulse frequency 20 Hz). **d** Graph shows the percentage of multi-joint complex movements during light stimulation. Stroke + ChR2-CREB produced a significant increase in multi-joint movements compared with stroke + ChR2-alone 4 weeks after stroke

If CREB induction in motor cortical neurons drives recruitment of these neurons into a larger cortical circuit that mediates recovery, we hypothesized that CREB-transfected neurons after stroke would control movement of greater body representations, extending over more of the motor map, than similarly treated neurons with a control virus. To test this hypothesis, we transfected CREB plus channel rhodopsin (ChR2) in a column of motor cortical neurons in the forelimb motor area (Fig. 4a). Blue light activation drives action potential activity with this viral construct (Fig. 4b). An optrode was used to activate this column of motor cortex neurons at 4 weeks after stroke (Fig. 4c), the time of motor recovery induced by CREB transfection. The number of forelimb vs multi-joint movements was quantified. CREB transfection in the forelimb area of normal (non-stroke) motor cortex increases the number of body movements evoked by local stimulation (Fig. 4d). Stroke causes a non-significant reduction in evoked movements from the forelimb motor cortex stimulation site. Stroke + lenti-CREB/ChR2 causes a significant gain in multi-joint movements compared with ChR2-stroke (Fig. 4d). These movements localized to body parts outside of the forelimb, in the CREB-induced mice, indicating that induction of CREB in motor cortex with ChR2, compared to just ChR2-alone, allows activation of broader, or more extensive circuits than normally are activated by the same neurons without CREB.

### CREB facilitates the reorganization of the sensory map

**Fig. 5:**
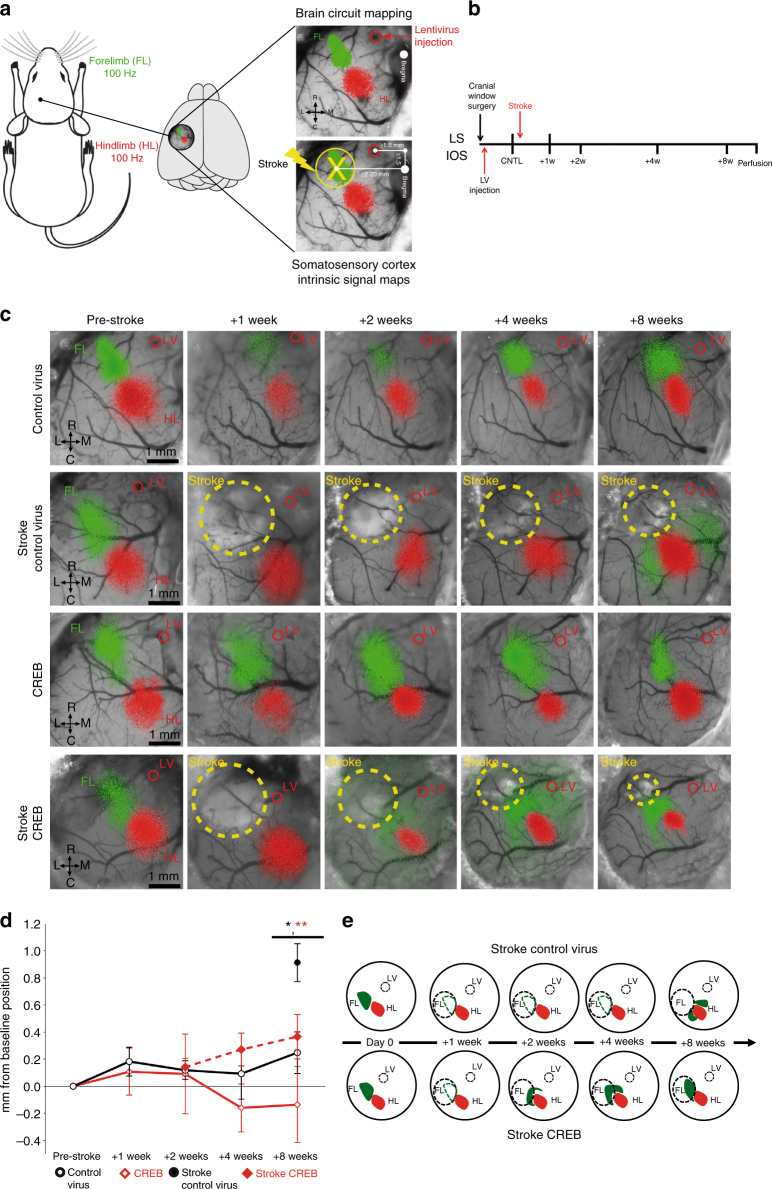
CREB promotes an early remapping of the sensory forelimb map after stroke. **a** Intrinsic optical signaling imaging (IOS) protocol. Piezoelectric stimulation (1 s, 100 Hz) of the sensory forelimb (FL) or hindlimb (HL) during IOS. **b** Experimental timeline shows IOS (baseline, +1 week, +2 weeks, +4 weeks, and +8 weeks), lentiviruses injection, and targeted stroke. **c** Time course and regional distribution of the IOS response maps to FL (green) and HL (red) stimulation in somatosensory cortex. IOS response maps were merged with an image of the surface vasculature to create regional maps of FL and HL activation. Stroke + CREB induction produced an early (over 2 weeks) recovery of the sensory forelimb map compared with stroke + Control virus (8 weeks). This reorganization occurs in the same regional map (baseline) with no displacement toward the motor forelimb representation. **d** Displacement of sensory forelimb (sFL) center from its mean baseline position before and after sensory stroke. Stroke + control virus causes a significant shift (1 mm) of the FL map (evident at 8 weeks) compared with stroke + CREB. ^♦♦^*P* < 0.001. Error bars represent SEM. *n* = 5 for all conditions **e** Schematic shows the difference in regional remapping and time course during which it occurs between stroke + control virus and stroke + CREB

The above data suggest that peri-infarct transfection of CREB accelerates recovery by increasing the recruitment of motor neurons into a functionally active motor recovery network. CREB also plays a key role in remapping of sensory functions in response to altered afferent input, such as in somatosensory cortex after whisker trimming^27,28^. During stroke recovery somatosensory maps move into motor cortex^29^, a result that indicates that stroke causes cortical remapping. It is possible that CREB affects this cortical remapping process. To test this hypothesis, fore- and hind-paw responses were mapped during stroke recovery (Fig. 5a). CREB or control viruses were injected into motor cortex as described above. In these studies, stroke was targeted to the forelimb somatosensory cortex, which is associated with a shift in forepaw representation into motor cortex during recovery. Chronic intrinsic optical signal (IOS) imaging was performed in the somatosensory cortex over 8 weeks after stroke (Fig. 5a, b). In the normal cortex, stimulation of the contralateral forelimb (FL) and hindlimb (HL) produces an IOS response in the somatosensory forelimb (sFL) and hindlimb (sHL) that is stable over time (Fig. 5c, d). Stroke caused a loss in this sensory forelimb response, which persisted over 4 weeks. Eight weeks after stroke, forelimb stimulation evoked a weaker forepaw response in the shifted region, including motor cortex and adjacent hindlimb somatosensory cortex (Fig. 5c, d). This spatial shift of forelimb somatosensory responses into motor cortex represents a significant effect within the sensory map (Fig. 5d), and has been previously reported^29,30^. No changes were observed to the hindlimb map for all the groups at any time point in all the groups (Supplementary Figs. 14, 15). IOS mapping of mice transfected with lenti-CREB showed an early recovery of the sensory forelimb map into the same position as the control forelimb somatosensory response, with no shift toward the motor forelimb map (mFL). This recovery with CREB occurs significantly faster (2 weeks after stroke) and is stable (Fig. 5c, d). The remapped representation of FL appeared in the same position as the control FL somatosensory response, with no shift toward the mFL. Importantly, the magnitude of the stroke was similar across animals infected with CREB or control lentivirus, as measured by laser speckle contrast microscopy (Supplementary Fig. 13). These results indicate that CREB induction in motor cortex facilitates the recovery of the sensory FL map after somatosensory stroke, by accelerating the time course of remapping into motor cortex (Fig. 5e). Sensorimotor recovery in humans is most significant when movement and sensory representations recover in their original or closely adjacent regions^31^. These data indicate that increasing CREB levels in peri-infarct cortex after stroke, establishes recovery in the appropriate somatosensory cortical representation, a mechanism thought to be key for successful recovery.

### CREB overexpression after stroke produces axonal sprouting

**Fig. 6:**
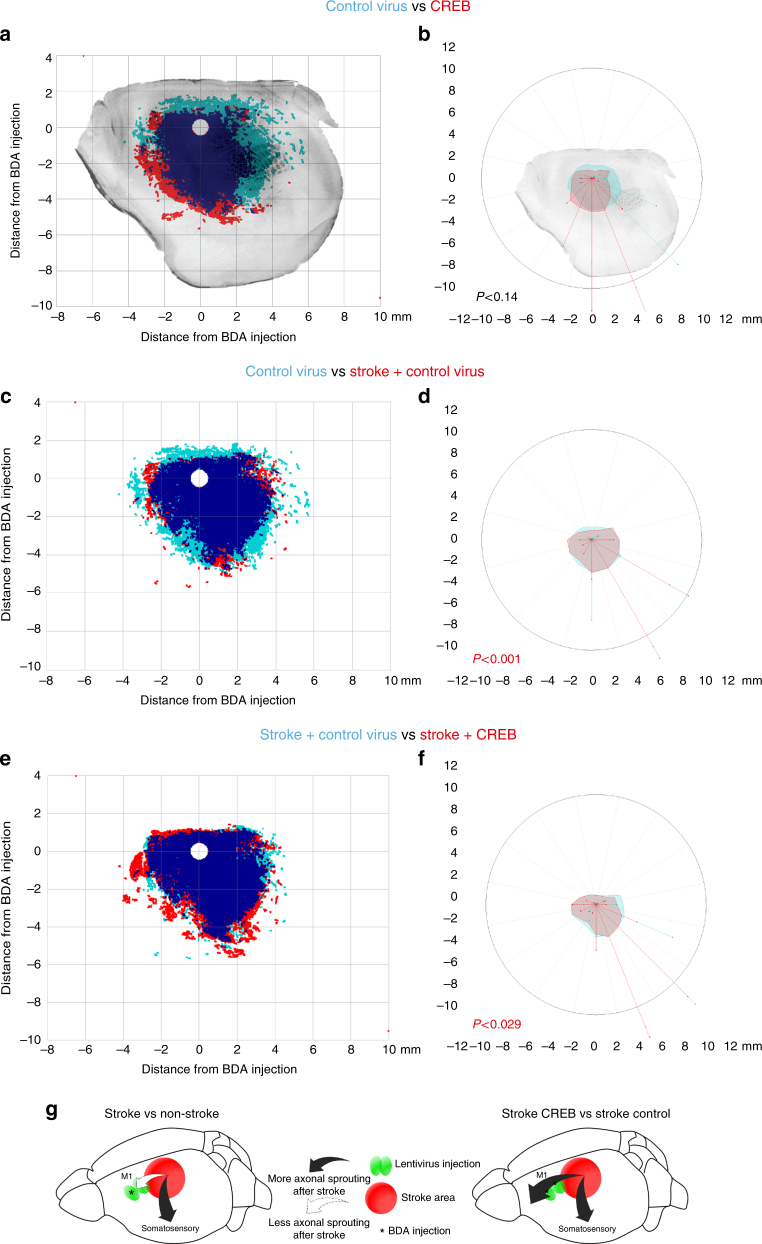
Quantitative mapping of motor system connections after stroke and with CREB induction. **a**, **c**, **e** Each map represents all of the digitally mapped connections from all the animals in each condition (*n* = 4 for each condition) from M1 anterior to the stroke site, collapsed onto a representative tangential section through the mouse cortical hemisphere. The *Y* and *X* axes show mm distance from the center of the tracer injection site. The dark blobs on the maps are the location of the primary somatosensory vibrissal field (the “barrel” field). Light blue label corresponds to the condition in the light blue labeled text at the top of each panel; red corresponds to the condition in the red label at the top of each panel; and dark blue is dense overlap. **b**, **d**, **f** In polar plots of connections of forelimb motor cortex projections the plot is made relative to the tracer injection in forelimb motor cortex as the origin. Each filled polygon (blue and red) is the 70th percentile of the distances of all BDA-labeled connections from the injection site in each segment of the graph. The lines in these plots are the median vector of that segment of the plot, multiplied by the median of the normal distribution of the number of points in a given segment of the graph. *P* value is Hotelling's *T*^2^. **g** Schematic summary of axonal sprouting after stroke with CREB induction

Stroke induces axonal sprouting within motor, pre-motor, and somatosensory cortical areas, and these new connections are correlated with functional motor recovery^17^^–^^19^^,^^32^. CREB drives axonal growth from neurons over inhibitory substrates in spinal cord^33^, suggesting that a mechanism for CREB action in motor recovery after stroke may be through axonal sprouting in motor circuits. The tracer BDA was microinjected into motor cortex 13 weeks after stroke, and animals were killed 1 week later (Fig. 2a). Axonal sprouting is identified when a pattern of cortical projections is precisely mapped, by digital tracing of each BDA-labeled projection, and is statistically different across treatment conditions^16–19^. The location of axonal connections in each mouse cortical hemisphere was plotted, the digital maps of each axonal projection were grouped by condition, and cortical projection maps were then quantitatively compared across treatment groups (Hotelling’s *t*^2^ test) for overall differences in cortical projections, and for specific areas that have a different pattern of connections as previously described (Fig. 6a–g).

New connections in peri-infarct cortex in stroke can be detected in both genetic approaches in delivery of CREB; in particular we observed a significant increase in BDA-labeled projections after stroke in motor cortex in lenti-CREB (*n* = 4, Hotelling’s *t*^2^ test *P* < 0.021) mice with stroke when compared, respectively, with their controls (CREB virus alone, Fig. 6c, d) or with the control virus (control (non-CREB) virus plus stroke, *P* < 0.029) (Fig. 6e, f). Lenti-CREB alone in the normal, non-stroke brain does not promote axonal sprouting (lenti-CREB alone vs control virus: *n* = 4, *P* = 0.25, Fig. 6a, b). These data indicate that induction of CREB in forelimb motor cortex produces axonal sprouting, particularly in motor-to-premotor connections, a pattern that is associated with functional recovery^16–19^.

### CREB induces a distinct transcriptional profile after stroke

**Fig. 7:**
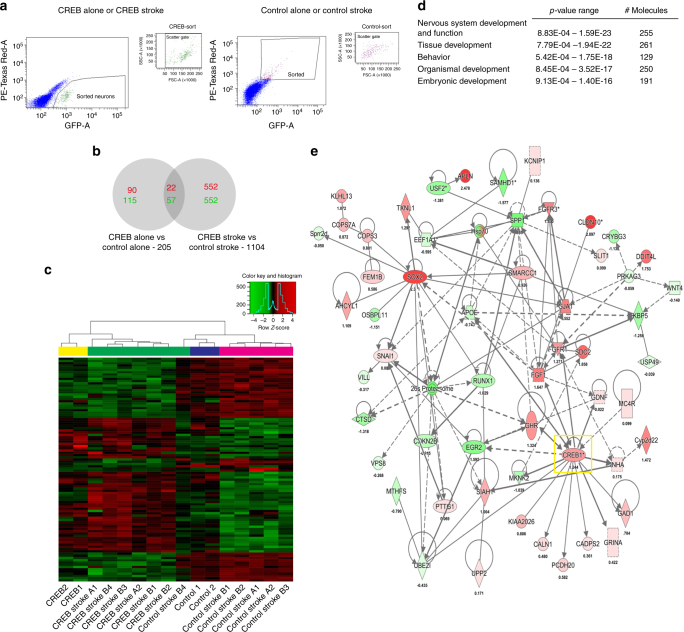
CREB-stroke transcriptome in motor cortex during period of recovery. **a** Fluorescence-activated cell sorting (FACS) isolation of CREB-transfected or control neurons in motor cortex after stroke. In the scattergrams, each selected cell is indicated by a dot. The cluster of sorted neurons (cells in the gate) in the scattergram corresponds to neurons that were selected for further analyses of their fluorescence characteristics. GFP-positive (CREB-transfected) or GFP-/Texas Red-positive cells (control virus, tdtomato-eGFP) with high fluorescence levels are displayed in a fluorescence scattergram where FSC (forward scatter light) represents the absorbance of transmitted laser light for each neuron and SSC (side scatter light) the light scattered at a 90-degree angle. **b** Hypergeometric overlap of genes that are significantly induced in control motor cortex neurons with CREB transfection compared to normal, non-transfected neurons that overlap with CREB-transfected neurons after stroke compared to non-transfected neurons after stroke. Red: upregulation; green: downregulation. There is little overlap of the two transcriptomes. **c** Unsupervised clustering of top 100 differentially expressed genes at *P* < 0.005. *Z* score is indicated by top right inset. **d** Top regulated canonical pathways in Stroke + CREB vs Stroke. Middle column shows the inverse log of the *P* value corrected for multiple comparisons in Benjamini–Hochberg (B–H) test. The left column shows the number of significantly regulated genes in the Stroke-CREB transcriptome that are within this canonical pathway. **e** Molecular pathway analysis showing interactions between significantly regulated genes in the Stroke-CREB transcriptome. Red is upregulated with darker indicating greater fold change. Downregulation is in green with same color strength scheme. Log2 fold change is below each gene

CREB modulates synaptic plasticity by altering gene expression and acts as a direct transactivator of regeneration-associated genes to mediate axonal sprouting^34^. To identify the molecular systems that are induced by CREB during behavioral recovery after stroke, CREB-transfected or control pyramidal neurons from motor cortex were FACS-isolated at the time of enhanced motor recovery (4 weeks after stroke) in control (non-stroke) and stroke conditions (Fig. 7a). RNA was isolated and used to probe whole genome arrays. To confirm this transcriptional profile, FACS isolation and microarray analysis were performed separately in the second cohort of Control Stroke and CREB Stroke animals (termed Control Stroke “A” and “B” and CREB Stroke “A” and “B”). Gene expression data were filtered to only include those with a gene expression *P*-value threshold of <0.005 and a minimum log2-transformed fold change of 0.2. Unsupervised clustering of the 100 most differentially regulated genes in each data set indicates that the condition with the greatest effect on gene transcription is induction of CREB or exposure to stroke (Fig. 7c): the transcriptional effect of CREB induction, stroke, and stroke + CREB drive the difference between transcriptomes in neurons. To understand how CREB induction in cortical neurons affects the normal transcriptional state induced by stroke, we compared Control Stroke across the two cohorts to Stroke CREB induction in the two cohorts. The transcriptional profile of Control Stroke clusters most closely to the replication study of Control Stroke, as expected (Fig. 7c). CREB induction in stroke produces a distinct transcriptome, with both initial and replication runs of CREB Stroke neurons having a similar transcriptional profile, and one that is distant from Control Stroke (Fig. 7c). This indicates that CREB induces a very distinct transcriptome within the condition of stroke as compared to inducing CREB in cortical neurons in the control state. In total, 205 genes were differentially expressed between CREB alone vs Control alone (90 up-regulated and 115 down-regulated genes, *P* value < 0.005) and 1104 genes were differentially expressed between Stroke CREB vs Stroke Control virus (552 up- and 552 down-regulated genes, *P* value < 0.005) (Fig. 7b). Gene ontology analysis in Stroke Control vs Stroke CREB shows that CREB induction in stroke activates sets of genes within cellular pathways that are relevant to neural repair and recovery, including nervous system development, tissue development and organismal development (Fig. 7d).

To identify specific molecular targets of CREB induction during the period of behavioral recovery after stroke, we analyzed the significantly regulated genes in the Stroke CREB transcriptome for co-regulated molecular pathways with known protein–protein interactions (Fig. 7e). CREB induction in stroke differentially regulates a distributed network of genes with roles in neuronal plasticity or recovery after stroke, including induction of the obligatory NMDA receptor subunit 1^35^ FGF, GDNF, and GH and their receptors^36^; the transcription factor Sox2^37^; Protocadherin 1^38^ and the calcium-binding proteins, calneuron 1^39^, and calcium-dependent secretion activator 2^40^. As CREB is a universally expressed transcription factor, these genes that are selectively regulated in cortical neurons in Stroke + CREB identify molecular targets for a more specific pharmacology to promote stroke recovery, in addition to an approach of targeting isoforms of CREB-modulating enzymes that are uniquely expressed in stroke-affected brain regions, such as phosphodiesterase inhibitors^41^.

## Discussion

The main findings of this study are: 1. Lentiviral CREB transfection in motor neurons anterior to the stroke site enhances recovery of the motor deficit. 2. This region of motor cortex is the same area in which axonal sprouting in motor and premotor circuits after stroke mediate recovery^17,18^, and in which a CREB-induced gene product, BDNF, is necessary for motor recovery^20^. 3. This CREB effect is specific to this small pool of neurons in motor cortex near the stroke site, as CREB induction in other regions of peri-infarct tissue does not alter recovery. 4. Blockade of CREB signaling inhibits normal motor performance after stroke. 5. Remarkably, inducibly and reversibly inactivating CREB-transfected motor neurons turns on and off motor recovery. 6. CREB induction accelerates the remapping of lost cortical sensory maps after stroke, with a timeline that matches that of accelerated functional recovery. 7. As a transcription factor, CREB in stroke activates a unique transcriptome of genes that play a role in neuronal excitability and developmental neuronal plasticity, establishing possible molecular mechanisms for the CREB effect in motor recovery.

Much of the focus of CREB signaling studies in stroke has been on the initial stages of ischemic cell death^42,43^. Later in stroke, there is no detectable difference in CREB activation in the recovering brain after stroke and the control, non-stroke brain^44^. pCREB and CRE-induced gene expression are seen with a maximum in the first 2 days after stroke^45^, although CREB activation can be detected in glial cells for weeks after stroke, where it has a role in neurogenesis and gliogenesis^46,47^. The present data indicate that treatments that activate CREB after the period of cell death may provide a target for a neural repair therapy in stroke.

The present findings have two important implications in the motor circuit control of recovery after stroke. First, motor recovery after stroke can be produced by modulating CREB signaling in a small pool of motor cortical neurons near the stroke site. This finding that CREB induction in a small subset of a brain circuit will change function of that circuit is supported by the effect of CREB in memory paradigms. During fear conditioning, roughly 70% of neurons in the lateral amygdala receive sensory inputs, but only about one-quarter exhibit learning-related synaptic plasticity^48–50^. CREB manipulation in approximately 20% of lateral amygdala neurons is sufficient to influence fear conditioning. In the present study, lentiviral transduction of CREB in ~9000 neurons, roughly 16% of the total motor cortex, is sufficient to induce motor recovery after stroke. This finding is the first to identify a specific motor circuit for recovery after stroke. How does this finding relate to the neuronal network architecture for movement control and does CREB activation expand a functional motor circuit for forelimb movement? In spontaneous limb movements, motor cortex is grouped into microcircuits of neurons with highly correlated activity that span 100 µm^51^, and with the same distance (70 µm) with a trained forelimb task^52,53^. In a different circuit measure, motor cortex input comes from within the local cortical circuitry and this has been estimated at 500 µm^54^. With this microcircuitry in mind, the lentivirus approach clearly transfects CREB in a large number of movement related motor cortex microcircuits. However, a key point from the present data is that, within these microcircuits, CREB transforms their function into a dominant role, such that inhibiting them significantly diminishes motor output to the contralateral forelimb, which does not occur if these circuits are inhibited but CREB is not induced.

A second implication of the present findings in motor networks for stroke recovery is that CREB transfection appears to disproportionally commit motor cortical neurons into a network for movement control both in normal brain and with a much larger effect after stroke. This is supported by three findings. First, CREB-transfected neurons are more likely to be activated in expressing immediate early genes during movement than neurons transduced with the same virus without CREB. Second, CREB-transfected motor cortical neurons in the motor forelimb area after stroke are more likely to activate body movements outside of that forelimb area. Third, acute inactivation of CREB-transfected motor cortical neurons produces motor control deficits, whereas acute inactivation of these neurons without CREB does not. This inactivation finding is distinct between control (non-stroke) and stroke. In the control (non-stroke) brain, inactivation of CREB-induced neurons in forelimb motor cortex impairs motor control but inactivation of a similar-sized pool of motor cortical neurons without CREB transfection does not impair motor control. In stroke, inactivation of CREB-transfected motor cortical neurons not only impairs motor control, as seen in the control (non-stroke) brain, but does so in a truly substantial way with a doubling of the stroke deficits, particularly in gait. Inactivation of motor cortical neurons after stroke that have not been transfected with CREB does not produce motor deficits on top of those of the stroke itself. For comparison, inactivation of CREB-transfected neurons has a much greater effect on memory recall than inactivation of a similar number of neurons that do not have CREB transfection^9,55^. These parallels between neuronal network alteration with CREB induction in motor cortex and in memory systems in the amygdala support a general role of CREB in committing neurons into an active circuit—in the impaired motor control after stroke and in the memory trace during a learning task.

These studies in stroke support a concept of two levels of plasticity in motor cortex: CREB-induced plasticity and Stroke-induced plasticity. In the first, the transcriptional effect of CREB allows a population of neurons in a region of motor cortex to exert greater control in movement of the body part represented in that network. This cortical circuit-capturing effect of CREB even in the normal brain could be done by increasing the number of neurons in a movement-associated ensemble^56^ or by increasing the correlated firing activity in the same ensemble^57^. Inactivation of CREB-transfected neurons in the normal brain in this first level of circuit plasticity produces a deficit in normal motor control—interestingly one that is equivalent in magnitude to that produced by stroke. In the second level of plasticity, stroke itself opens up plasticity and a process of change in cortical maps^27,28^, and CREB-induced neurons build on the post-stroke plastic state to drive control of even more of the motor cortical circuit. In this post-stroke state, inactivation of CREB-transfected neurons more profoundly impairs motor control—much more than stroke alone. The mechanisms for the first level of plasticity—CREB-induced plasticity—are likely the CREB-produced changes in cellular excitability and preferential commitment of CREB neurons into a motor circuit, as in memory systems^10–12^. The mechanisms for the second level of Stroke-induced plasticity are not clear, but several excitability changes occur in this region of peri-infarct cortex. Tonic GABA signaling is increased^8,9^, which depresses pyramidal neuron excitability. This may provide for a greater delta in the excitability increase with CREB and in secondary synaptic integration of a CREB-transfected neuron. Excitatory signaling through the AMPA receptor in this region of peri-infarct cortex shows enhanced sensitivity to the induction of BDNF^20^, suggesting that a further CREB-induced increase in neuronal excitability might result in even greater downstream plasticity effects. As noted, motor cortical neurons that control a voluntary limb movement after stroke form functional clusters that are likely the basis for CREB-induced plasticity and Stroke-induced plasticity.

Expression of dominant-negative CREB in the primary visual cortex prevents ocular dominance plasticity, suggesting that CREB function may underlie the competitive interaction responsible for axonal territory assignment in the developing visual system^13,14^. CREB is necessary for the experience-dependent plasticity that underlies cortical responses to peripheral lesions in the somatosensory cortex^27^. The present data indicate that CREB also plays a role in the plastic remapping of cortical representations after stroke. Stroke damage eliminates the somatosensory body map, which then reforms after a 4-week delay in adjacent motor and ectopic somatosensory regions^29,32^. CREB induction accelerates the timeline during which injured cortex can remap somatosensory representations and the nature of this remapping: the forepaw somatosensory cortex is remapped in very close proximity to its original location (Fig. 5d). This pattern of remapping also matches the distribution of axonal projections in peri-infarct cortex (Fig. 6b, d, f). In human stroke, remapping of sensorimotor functions into sites close to their original location is associated with greater recovery^31^. Just as CREB plays a key role in regulating visual cortex allocation of eye-specific inputs, the present data indicate that after stroke CREB plays a key role in regulating somatosensory allocation of limb-specific inputs during recovery.

## Methods

### Animals

All procedures were performed under an NIH approved animal protocol and the University of California Los Angeles Chancellor’s Animal Research Committee. 2–4 month-old adult C57BL/6 (Charles River and Jackson Lab) or LBD-CREB male mice^23^ were maintained on a 12 h light/dark cycle with free access to food and water.

### Photothrombotic model of focal cortical stroke

Under isoflurane anesthesia (2–2.5% in a 70% N_2_O/30% O_2_ mixture), mice were placed in a stereotactic apparatus, the skull exposed through a midline incision, cleared of connective tissue and dried. A cold light source (KL1500 LCD, Carl Zeiss MicroImaging, Inc.) attached to a 40× objective giving a 2-mm diameter illumination was positioned 1.5 mm lateral from Bregma, and 0.2 ml of Rose Bengal solution (Sigma; 10 g l^−1^ in normal saline, i.p.) was administered. After 5 min, the brain was illuminated through the intact skull for 15 min^16–20^.

### Model of combined cortical, subcortical white matter, and striatal stroke

The above procedure for photothrombotic stroke was followed. A 10 min illumination through the skull was followed by ipsilateral carotid ligation and 2 µl injection of the vasoconstrictor N5-(1-Iminoethyl)-L-ornithine (L-NIO; 27 mg/ml in sterile physiological saline; EMD Millipore) into the striatum (A/P 0.00, M/L 3.00, D/V 2.60; at 10^o^). After completion of the L-NIO injection the needle was withdrawn. To assess the size and location of this cortical/subcortical stroke, three mice were euthanized at 2 days post-stroke and processed for TTC staining.

### Cresyl violet stain

Cresyl violet stain was performed by immersing sections in 50, 70, 95, and 100% ethanol for 1 min each followed by 45 min immersion in 50% ethanol/50% chloroform. Slides were then immersed back through the 100, 95, 70, and 50% ethanol and rinsed in distilled water before being stained in cresyl violet solution for 45 s. Slides were rinsed in distilled water and dehydrated in 95 and 100% ethanol before being placed in xylenes and coverslipped.

### Infarct size analysis

Cresyl violet-stained sections from behavior-tested animals were imaged using a confocal microscope (Nikon C2). Cortical and hemispheric areas were traced using ImageJ (NIH). Percent cortical loss was measured by dividing ipsilateral cortex area by contralateral cortex area. Data was analyzed using a paired *t*-test.

### Lentivirus injection

Lentivirus injections were performed immediately after stroke (Fig. 1a). For the gain of function studies, we transfected the peri-infarct motor cortex of WT mice (anterior to the stroke lesion) with 1.5 µl of lentivirus that overexpress CREB (CamkIIa_HA_AlstR_F2A_EGFP/CREB) or the control lentivirus (CamkIIa_HA_AlstR_ F2A_EGFP/dTomato) in two different points (M/L: −1.5 mm, A/P: 1.0 and 1.5 mm, D/V: 0.75 mm) or we transfected the lenti-CREB immediately posterior (M/L: −1.5 mm, A/P: −2.1 mm, D/V: 0.75 mm) to the stroke lesion (PTLp^22^).

For the selective loss of function study, we transfected the peri-lesion motor cortex of mice with 1.5 µl of hM4Di-CREB (CamkIIa_HA/hM4Di_T2A_EGFP/CREB) or the control lentivirus (CamkIIa_HA/hM4Di_T2A_EGFP/dTomato)^24^ in the same area of the motor cortex (anterior to the stroke lesion; M/L: −1.5 mm, A/P: 1.0 and 1.5 mm, D/V: 0.75 mm).

### Tamoxifen administration

For the loss of function studies, groups of LBD-CREB mice were administered with TAM (16 mg/kg i.p., Sigma #T5648) or vehicle (similar volume of 0.9% saline solution) 6 h before behavioral tasks^23^.

### CNO administration

For the selective loss of function study, groups of C57BL/6 mice were administered with CNO (0.3 mg/kg i.p., Biomol International) or vehicle (similar volume of 0.9% saline solution) 30 min before each behavioral task.

### Behavioral assessment

Mice (8–10 per group) were tested on the grid-walking, cylinder, and capellini tasks, 1 week before surgery to establish baseline performance levels^17,18,20,21^. For the capellini task, mice were trained for a period of 2 weeks and subsequently animals were tested on weeks 1, 3, 5, 7, and 11 after stroke (Figs. 2–3; Supplementary Figs. 5–7, 9). Behaviors were scored by observers who were blind to the treatment or experimental group. These behavioral tests rely on a degree of exploratory behavior and novelty; more frequent testing than every 3 weeks (grid and cylinder) or 2 weeks (pasta handling) produces acclimation and lack of movement.

### Immediate early gene quantification

Mice received stroke and either (CamkIIa_HA_AlstR_F2A_EGFP/CREB) or the control lentivirus (CamkIIa_HA_AlstR_ F2A_EGFP/dTomato). Four weeks later, mice were euthanized, perfused with 4% paraformaldehyde, and the brains isolated and post-fixed overnight. 50 μm sections were cut and collected at every 200 μm intervals and stained immunohistochemically for phosphoCREB (pCREB, antibody source) or Zif268. Images were collected at 20× (Nikon C2 confocal) and co-localization of green fluorescence with red fluorescence used to visualize NeuN or red fluorescence in control virus and green fluorescence used to visualize NeuN was counted. Data was analyzed using a paired *t*-test.

### Electrophysiological recording and stimulation

Mice were deeply anesthetized with isoflurane and perfused with ice-cold high sucrose solution containing the following (in mM): 280 sucrose, 2.5 KCl, 1.25 NaH_2_PO_4_, 26 NaHCO_3_, 1.3 MgCl_2_, 8 MgSO_4_, and 10 glucose. Coronal slices (300 μm) were obtained using a microslicer (Leica VT1000S; Leica Microsystems) and transferred to an incubating chamber containing ACSF (130 NaCl, 3 KCl, 1.25 NaH_2_PO_4_, 26 NaHCO_3_, 2 MgCl_2_, 2 CaCl_2_, and 10 glucose) oxygenated with 95%O_2_–5%CO_2_ (pH 7.2–7.4, 290–310 mOsm). The slices were incubated for 40 min at 32 °C and more 20 min at room temperature. Pyramidal cells were visualized with a microscope (Olympus BX51WI), which was equipped with differential interference contrast optics and fluorescence. The recordings were obtained using a MultiClamp 700B Amplifier (Molecular Devices) and pCLAMP 10.5. The patch pipette (3–4 MΩ impedance) contained a Cesium methanesulfonate-based internal solution (in mM): 130 Cs-methanesulfonate, 10 CsCl, 4 NaCl, 1 MgCl_2_, 5 MgATP, 5 EGTA, 10 HEPES, 5 GTP, 10 phosphocreatine, and 0.1 leupeptin. In cell attached mode, blue light stimulation (CooLED, 473 nm, 4 mW) was used to activate ChR2-expressing neurons. The blue light (CooLED, 473 nm, 4 mW) evoked action potentials in the recorded cells. Yellow light (CooLED, 572 nm, 20 mW) did not trigger any response in the same cells (data not shown). The whole-cell patch-clamp recordings were done in voltage clamp mode. AMPA and NMDA receptor antagonists (NBQX 10 μM, APV 50 µM, Sigma) were added to the external solution.

### In vivo optogenetic stimulation of the motor cortex

Under isoflurane anesthesia immediately after stroke suspension of two lentiviruses, Lenti-CREB (1 μl) and Lenti-ChR2 (1 μl), were injected stereotaxically in the motor cortex M1 of C57BL/6 male mice in each condition (*n* = 5 Lenti-ChR2 control, *n* = 5 stroke plus Lenti-ChR2, *n* = 4 Lenti-CREB + Lenti-ChR2, and *n* = 4 stroke plus Lenti-CREB and lenti-ChR2). The injection coordinates were 1.5 mm lateral, 1 and 1.5 mm anterior, and 0.75 mm ventral to the Bregma. An optrode (250 micron core, fiber length 0.5 mm with 10% of light pass by, UCLA Behavioral Testing Core) was positioned stereotaxically between the two injections sites (M/L: −1.5 mm, A/P: 1.25 mm, D/V: 0.5 mm). Titanium head bar was attached to the skull using a layer of dental cement. Over the next week, mice were head-fixed when awake and trained on a free-floating foam ball before optogenetic stimulation. Four weeks after stroke and lentiviruses injection, mice were videotaped and blue light-stimulated (*λ* = 473 nm) for 10 s (pulse length 5 ms, pulse frequency 20 Hz). The number of multi-joint movements (forelimb movements + mouse-wisker-trunk movements) was quantified (Fig. 4). A trunk movement is when the animal engages in a lateral or torsional movement of the axial musculature between the upper and lower extremity.

### Intrinsic optical signaling imaging

Chronic glass-covered cranial windows were implanted^16^. Briefly, mice were anesthetized with isoflurane (1.5% via nose cone) and placed in a stereotaxic frame over a warm water re-circulating blanket. Dexamethasone (0.2 mg/kg; Baxter Healthcare Corp.) and carprofen (5 mg/kg; Pfizer) were administered subcutaneously to reduce brain edema and local tissue inflammation. A 4 mm craniotomy was performed with a pneumatic dental drill. The center of the craniotomy was placed of over the left hemisphere, 3 mm lateral to the midline and 1.7 mm caudal to Bregma. A sterile 5 mm glass coverslip (Electron Microscopy Sciences) was gently laid over the dura mater and glued to the skull with cyanoacrylate-based glue. Dental acrylic was then applied throughout the skull surface. A titanium bar (0.125 × 0.375 × 0.05 inch) was embedded in the dental acrylic to secure the mouse on to the stage for imaging. Virus injection was carried out right after the opening of the skull (Fig. 5).

IOS imaging of the FP and HP sensory receptive fields was done at different intervals before and after RBPT stroke (control, 1 week, 2 weeks, 4 weeks, and 8 weeks). IOS imaging was performed through the cranial window on mice under light anesthesia with 0.5–0.75% isoflurane and a single dose of chlorprothixene (6 mg/kg, i.p., Sigma-Aldrich). The cortical surface was illuminated by green (535 nm) and red (630 nm) sets of light-emitting diodes (LEDs) mounted around a “front-to-front” tandem arrangement of objective lenses (135 mm and 50 mm focal lengths, Nikon). The green LEDs were used to visualize the superficial vasculature and the red LEDs were used for IOS imaging. The microscope was focused to ~350 µm below the cortical surface. Imaging was performed at 10 frames per second using a fast camera (Pantera 1M60, Dalsa), frame grabber (64 Xcelera-CL PX4, Dalsa) and custom routines written in MATLAB. Each session consisted on 30 trials, taken 20 s apart, of mechanical stimulation for 1.5 s (100 Hz) using a glass micropipette (blunt tip for) coupled to a piezo bender actuator (Physik Instruments). Frames 0.9 s before onset of stimulation (baseline) and 1.5 s after stimulation (response) were collected. Frames were binned three times temporally and 2 × 2 spatially. Stimulated cortical areas were identified by dividing the response signal by the averaged baseline signal (*DR/R*) for every trial and then summing all trials. Response maps were then thresholded at 50% of maximum response to get the responsive cortical areas for FP and HP. For figures, we aligned the response maps for FP and HP stimulation within and across animals for all time points (pre-stroke, +1 week, +2 weeks, +4 weeks, and +8 weeks) with the help of the corresponding photomicrographs of the superficial vasculature. To generate the final image, we merged and color-coded the responses for FP and HP (green and red, respectively) for every time point into a single RGB image in Adobe Photoshop (Adobe Systems Inc.) (Fig. 5; Supplementary Figs. 10, 11).

### Laser speckle contrast microscopy of cerebral blood flow

The cortical surface was illuminated with an expanded laser diode beam (785 nm, 80 mW, Thorlabs Inc.) coupled to a 600 µm diameter fiber optic cable (Thorlabs Inc.). Sequences of 100 images were acquired at 30 frames per second using a fast camera (Pantera 1M60, Dalsa) with 1024 × 1024 pixels yielding images of 3.25 × 3.25 mm^2^. Speckle contrast images (*K* = standard deviation/mean, 5 × 5 pixel area, 3.15 µm/pixel) for every frame were obtained and averaged over the 100 frames using a custom written ImageJ plugin (courtesy of Timothy Murphy, University of British Columbia, Vancouver, Canada). Relative cortical blood flow values were obtained as the ratio *K*_0_^2^/*K*_t_^2^ (Supplementary Fig. 12).

### BDA injection

For the stereological quantification of axonal sprouting, 12 week post-stroke animals were injected with the neuroanatomical tracer 10% biotinylated dextran amine (300 nl of BDA; 10,000 MW; Invitrogen) in the same position of lentivirus injection (M/L: −1.5 mm, A/P: 1.0 and 1.5 mm, D/V: 0.75 mm). One week later, mice were sacrificed and brains were sliced tangentially (40 μm)^16–19^. BDA was visualized in the same sections using the Standard Vectastain Elite Kit (Vector Labs) and the chromogen diaminobenzamidine.

### Quantification of axonal sprouting

Axonal sprouting was quantified as previously described^16–19^. Briefly, axonal sprouting (*n* = 4 animals per each condition: Control virus, stroke control virus, CREB, and stroke CREB) was quantified by digitally marking each BDA-positive process in the superficial layers of the cortex (layers 2/3 and 4) using a microscope system (Leica Microsystems) and analysis program (Stereoinvestigator, MBF Biosciences). BDA-positive processes were marked *x*/*y* coordinates relative to the center of the injection site by an observer blinded to the treatment conditions. This process generates an *x*,*y* plot of the location of all labeled axons in each brain section. The *x*/*y* axonal plots from each brain were registered with respect to the injection site and co-registered with functionally relevant anatomical regions, produced by the staining of the mouse somatosensory body map in cytochrome oxidase, to generate a composite projection map for each treatment condition. Custom software produces quantitative connectional maps that consist of pixels, with the number of axons in each pixel mapped in register with anatomical brain structures. Polar plots were constructed with the *x*,*y* position of each BDA-labeled element plotted in relation to the tracer injection in forelimb motor cortex as the origin. This polar mapping shows both location and direction of axonal label. Scatter plots and polar maps were analyzed for statistically significant differences in connectional profiles between treatment groups using Hotelling’s *t*^2^ test for spatial correlation^16–19^ (Fig. 6).

Axonal sprouting was analyzed in two cohorts, using the Lenti-CREB and Lenti-control viruses in stroke and control conditions and in a second independent study using the DREADD viruses (*n* = 5 animals per each condition: hM4Di/CREB plus saline, stroke hM4Di/CREB plus saline, hM4Di/CREB plus CNO, stroke hM4Di/CREB plus CNO, hM4Di virus plus CNO, and stroke hM4Di plus CNO).

### Isolation of neurons for fluorescence-activated cell sorting

Four-month-old male C57BL/6 mice were anesthetized with isoflurane, decapitated, and cortical tissue removed from underlying white matter. We used the same lentivirus from the gain of function study for lenti-CREB (CamkIIa_HA_AlstR_F2A_EGFP/CREB) and lentivirus control (CamkIIa_HA_AlstR_F2A _EGFP/tdTomato). We performed FACS isolations on two different cohorts of mice to examine cell-type-specific gene expression in mouse brain tissue. In the first study, we isolated eGFP+ cells (CREB-transfected neurons) or eGFP/tdTomato+ cells (Control virus transfected neurons) from a pool of three cortices of each treatment condition, following 4 weeks after stroke and lentivirus injection (samples: *n* = 2 for stroke control virus referred as Control Stroke A1 and A2, *n* = 2 for stroke CREB referred as CREB Stroke A1 and A2, *n* = 2 for control virus referred as Control1 and Control2, *n* = 2 for control CREB referred as CREB1 and CREB2) (Fig. 7c). In the second study, we isolated eGFP+ cells or eGFP/tdTomato+ cells from a pool of three cortices, 4 weeks after stroke and viral transfection, from stroke control virus (*n* = 4 samples; Control Stroke B1–B4) and stroke CREB (*n* = 4 samples, CREB stroke B1–B4) (Fig. 7c). Consequently, these two different cohorts of FACS-isolated neurons were used to generate double-stranded DNA for two different Mouse Ref 8 v 2.0 Gene Expression chip (Illumina) (Fig. 7c). Peri-lesion cortices area corresponding to the lentiviral injection site (1 mm^2^, anterior to the stroke site) from control or stroked-mice (CREB-transfected, stroke CREB-transfected, control virus transfected, or stroke control virus transfected; 4 weeks after lentivirus injection and stroke) were dissected using a scalpel. Cortical tissue was enzymatically digested and triturated^20^. Briefly, cortical tissue was equilibrated for 8 min and digested for 30 min at 30 °C and 190 r.p.m. in 6 ml of papain solution (12 mg per ml). Complete Hibernate buffer (Brainbits) was used to maintain neural metabolites and pH during tissue dissection and digestion. Tissue was triturated into 6 ml of suspension and loaded onto density gradient column (4 ml of 12.4% OptiPrep in Hibernate), and centrifuged for 15 min at 900×*g* at 22 °C. The bottom 5 ml was collected and washed twice at 400×*g* for 5 min. Three cortices were pooled for each group (stroke control virus, stroke CREB, control virus, and control CREB) for FACS analysis (Fig. 7). Samples were maintained on ice during FACS isolation. APC sort gates were set using positive and negative controls before neuron sorting. Neurons were collected via FACS (FACsARIA, Becton Dickinson, UCLA FACS Core) directly into 400 μl of lysis buffer for RNA isolation. Total RNA was extracted using RNA-Microprep kit (Zymo-Research) and eluted into 7 μl ddH_2_O. RNA quality was verified (RIN > 7) on an Agilent Bioanalyzer.

### RNA preparation and array hybridization

Total RNA from FACS-isolated cells from each group was pre-amplified and converted into double-stranded DNA using Ovation PicoSL WTA System v2 (Nugen Technology) and biotinylated using Encore BiotinIL Module (Nugen Technology) prior hybridization (UCLA Neuroscience Genomic Core, UNGC) on Mouse Ref 8 v 2.0 Gene Expression chip (Illumina) according with Nugen Technology protocol.

### Microarray analysis

Raw data were analyzed using the EdgeR Bioconductor package54, and differentially expressed genes were classified according to gene ontology based on a *P* value, *P* < 0.005. Differentially expressed genes were analyzed by molecular pathway analysis and canonical signaling systems using Ingenuity Pathway Analysis software (IPA, Ingenuity Systems) (Fig. 7). We performed two separate FACS isolations and microarray analysis from different cohorts of mice. In the first study, we pooled three cortices for each condition following 4 weeks after stroke (*n* = 2 for stroke control virus, *n* = 2 for stroke CREB, *n* = 2 for control virus, and *n* = 2 for control CREB) (Fig. 7). In the second study, we pooled three brains for each condition (*n* = 4 for stroke control virus and *n* = 4 for stroke CREB). No differences were observed between the two studies.

### Histology

One week after stroke and/or lentiviral injection, mice were killed by transcardial perfusion with 4% paraformaldehyde in 0.1 M phosphate buffer (pH 7.4, wt/vol). Brains were postfixed in 4% paraformaldehyde overnight, cryoprotected in 20% sucrose, frozen, and sliced coronally (40 μm). A series of sections 200 μm apart was incubated with NeuN (to label neurons, 1:100, Millipore, #MAB377), GAD67 (to label inhibitory neurons, 1:800, Millipore, #C265), GFAP (to label astrocytes, 1:1000, ThermoFisher Scientific, #13–0300), and Glucose Transporter-1 (Glut-1, to label blood vessels, 1:1000, Millipore, #400060) (Fig. 1; Supplementary Figs. 2–4). Free-floating sections were rinsed, blocked in 3% normal donkey serum and 0.3% Triton for 1 h, incubated in primary antibody in 3% normal donkey serum and 0.1% Triton overnight at 4 °C, rinsed, incubated in secondary antibody [Dylight 488F(ab)2 or Dylight 597F(ab)2 anti-rabbit, anti-rat, or anti-mouse; Jackson Immunoresearch] in 0.1% Triton for 1 h, rinsed, mounted, and cover-slipped. Imaging was performed using a Nikon C2 confocal laser scanning microscope. The images were taken using a 20× and 60× objectives. The total number of CREB/eGFP-positive cells in the peri lesions motor cortex was counted across at least six sections from comparable antero-posterior levels from each mouse. The percentage of CREB/eGFP-positive cells was calculated as the percentage of CREB/eGFP-positive cell per total NeuN-labeled cells in the motor cortex.

For the immediate early gene study (Fig. 3), 4 weeks after lentiviral injection brains were sliced coronally (40 μm) and incubated with Zif268 (Zinc finger protein 225 or erg-1; 1:750, Cell Signaling, #4153S) or p-CREB (phospho-CREB, 1:800, Cell Signaling, #9198).

Automatic counting in multiple fluorescent images was performed using ImageJ software, version 1.4 (National Institutes of Health). Statistical analysis was performed using paired two-tailed Student *t* tests.

### Statistical analysis

Mice were randomly allocated to treatment condition using a randomized block experimental design (restricted randomization) and all results were analyzed with the investigator blinded to treatment condition. No animals were excluded from analyses. Differences between two means were assessed by unpaired two-tailed Student’s *t* test. Data from behavioral experiments were analyzed by two-way repeated-measures ANOVA followed by Bonferroni’s post hoc test. Data from the optogenetic study were analyzed by multiple comparisons 1 ANOVA followed by Kruskal–Wallis’s test. All statistical analyses were performed with GraphPad Prism version 6 (GraphPad Software). The level of statistical significance was set at *P* *<* 0.05. All data are expressed as mean ± SEM. Sample size in tissue outcome and behavioral studies was assessed by power analysis using a significance level of *α* = 0.05 with 80% power to detect differences in ANOVA.

Animal number in in vivo quantitative cortical mapping studies utilizes spatial correlation statistics, so sample size was estimated from previous publications with similar mechanistic studies^16–20^. Scatter plots were analyzed using Hotelling’s *t*^2^ test for spatial correlation^16–20^.

### Data availability

The datasets generated during and/or analyzed during the current study are available from the corresponding author on reasonable request.

## Electronic supplementary material

Supplementary Information

Peer Review File
