# Supplementary material for: CREB controls cortical circuit plasticity and functional recovery after stroke
Source: Nat Commun. 2018 Jun 8;9:2250. doi: 10.1038/s41467-018-04445-9 (PMC5993731; doi:10.1038/s41467-018-04445-9)
Supplement: Supplementary file 1 — Supplementary Information [file 41467_2018_4445_MOESM1_ESM.pdf]

CREB Controls Cortical Circuit Plasticity and Functional Recovery after Stroke

Caracciolo et al.

Supplementary Figures and Legends

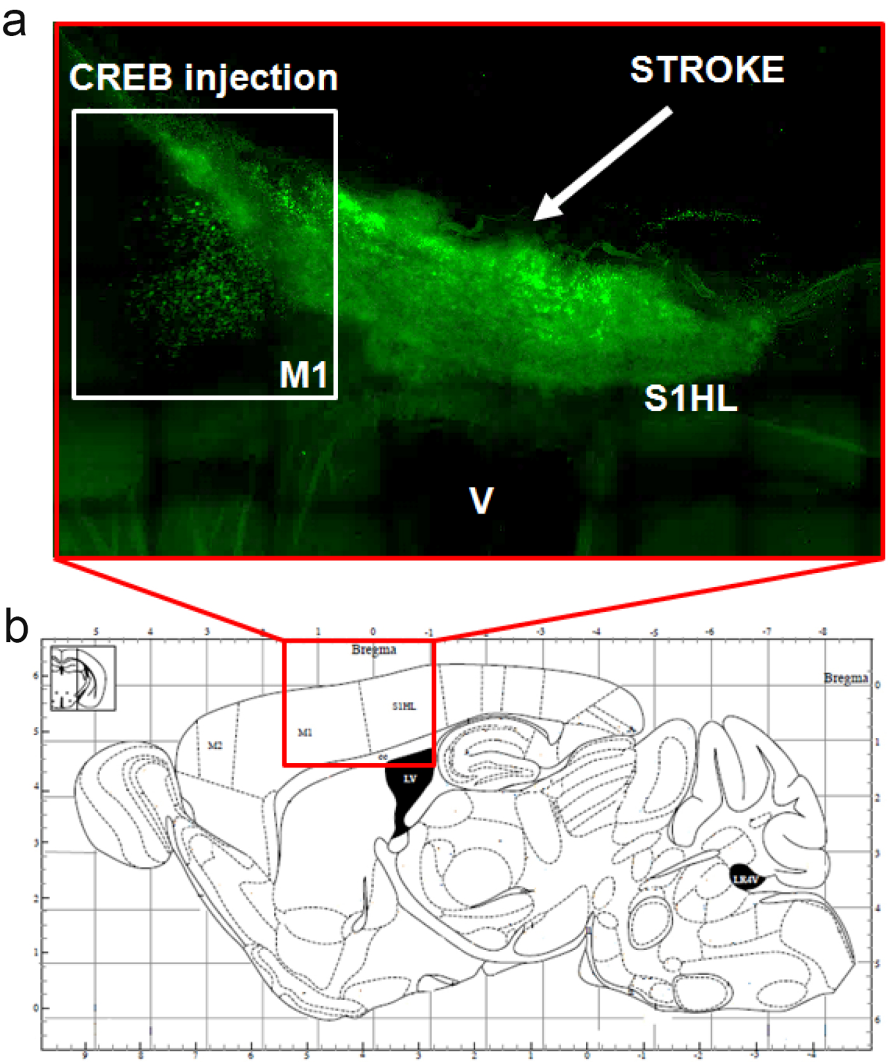
a
CREB injection
STROKE
M1
S1HL
V
b
Bregma
M2
M1
S1HL
LV
Bregma

Suppl Fig 1

Supplementary Figure 1. Lentivirus injection sites and motor cortex stroke. (**a**) Image of sagittal section cleared with the Clarity technique showing endogenous background green fluorescence from debris in the necrotic core of the stroke (arrow) and GFP fluorescent cells labeled by lentivirus (box). Anterior is to left, and ventral is to bottom of image. (**b**) Sagittal image from mouse atlas (ref 23 in text) depicting location of (a). M1 = primary motor cortex. S1HL = hindlimb area of primary somatosensory cortex. v = ventricle. In (b), LV = lateral ventricle, M2 = second motor area, cc= corpus callosum.

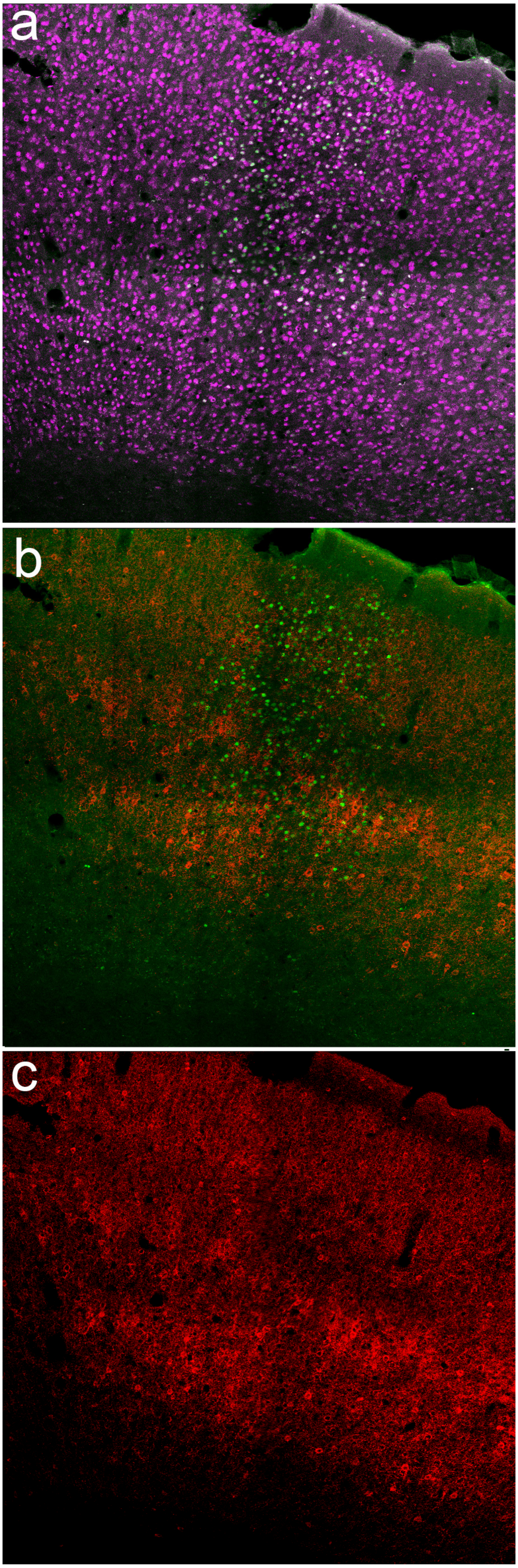

Suppl Fig 2

Supplementary Figure 2. Lentiviral transfection of neurons in primary motor cortex. (**a**) NeuN immunohistochemical stain (purple) and lentiviral transduced cells (green). Pial surface of cortex is to top and white matter surface of cortex is at bottom. (**b**) GAD67 (red) immunohistochemical staining of same section as (a). Few lentiviral cells are GAD67+. (**c**) GAD67 immunohistochemistry only. See also Fig. 1E.

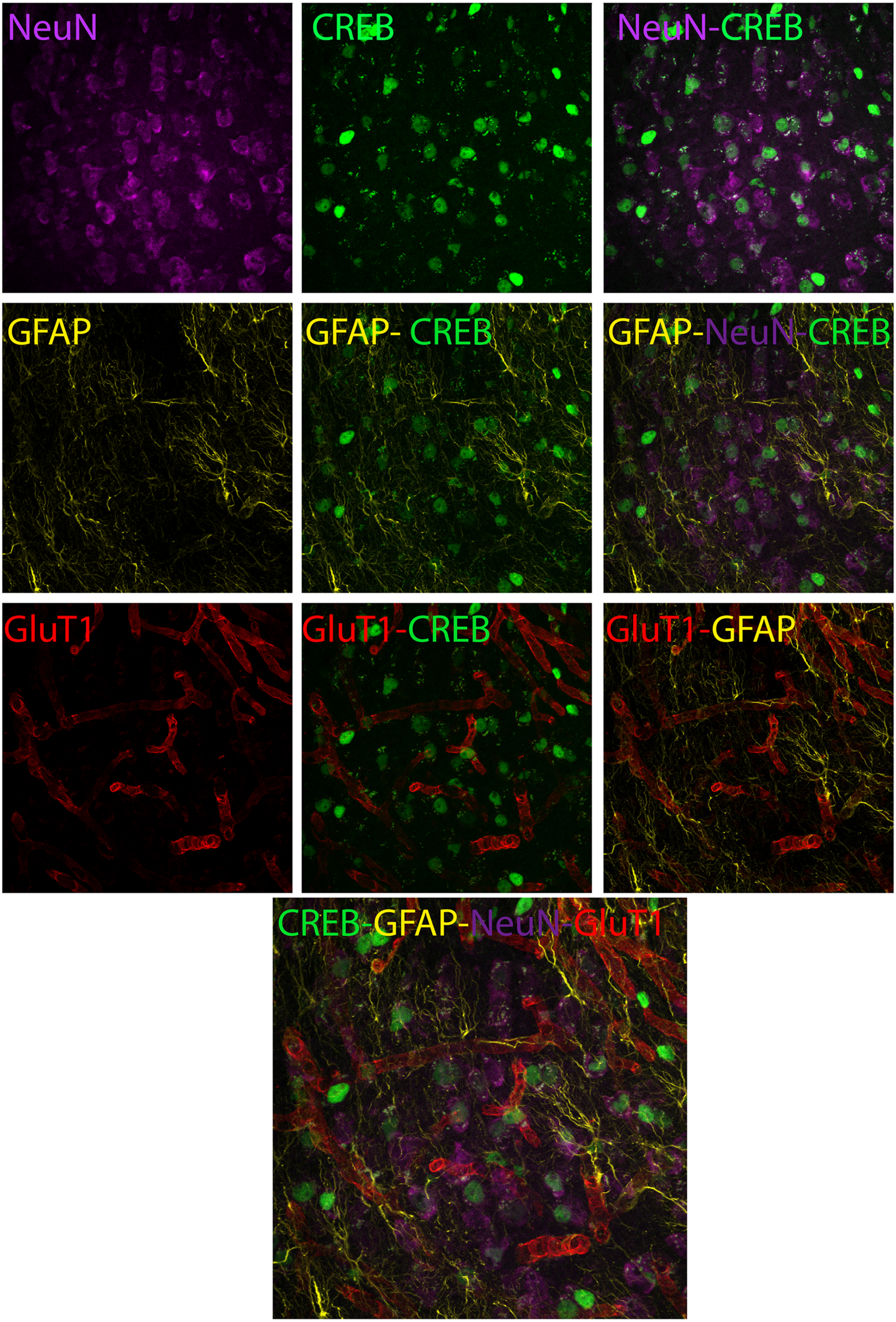
NeuN
CREB
NeuN-CREB
GFAP
GFAP- CREB
GFAP-NeuN-CREB
GluT1
GluT1-CREB
GluT1-GFAP
CREB-GFAP-NeuN-GluT1

Suppl Fig 3

Supplementary Figure 3. Lentiviral CREB transfection 1 week after stroke. All images are from one section with multi-fluorescent confocal imaging of neurons (NeuN, purple), astrocytes (GFAP, yellow), and blood vessels (Glut-1, red). CREB is localized as a GFP fusion protein in the nucleus. CREB co-localizes with neurons but not astrocytes or blood vessels.

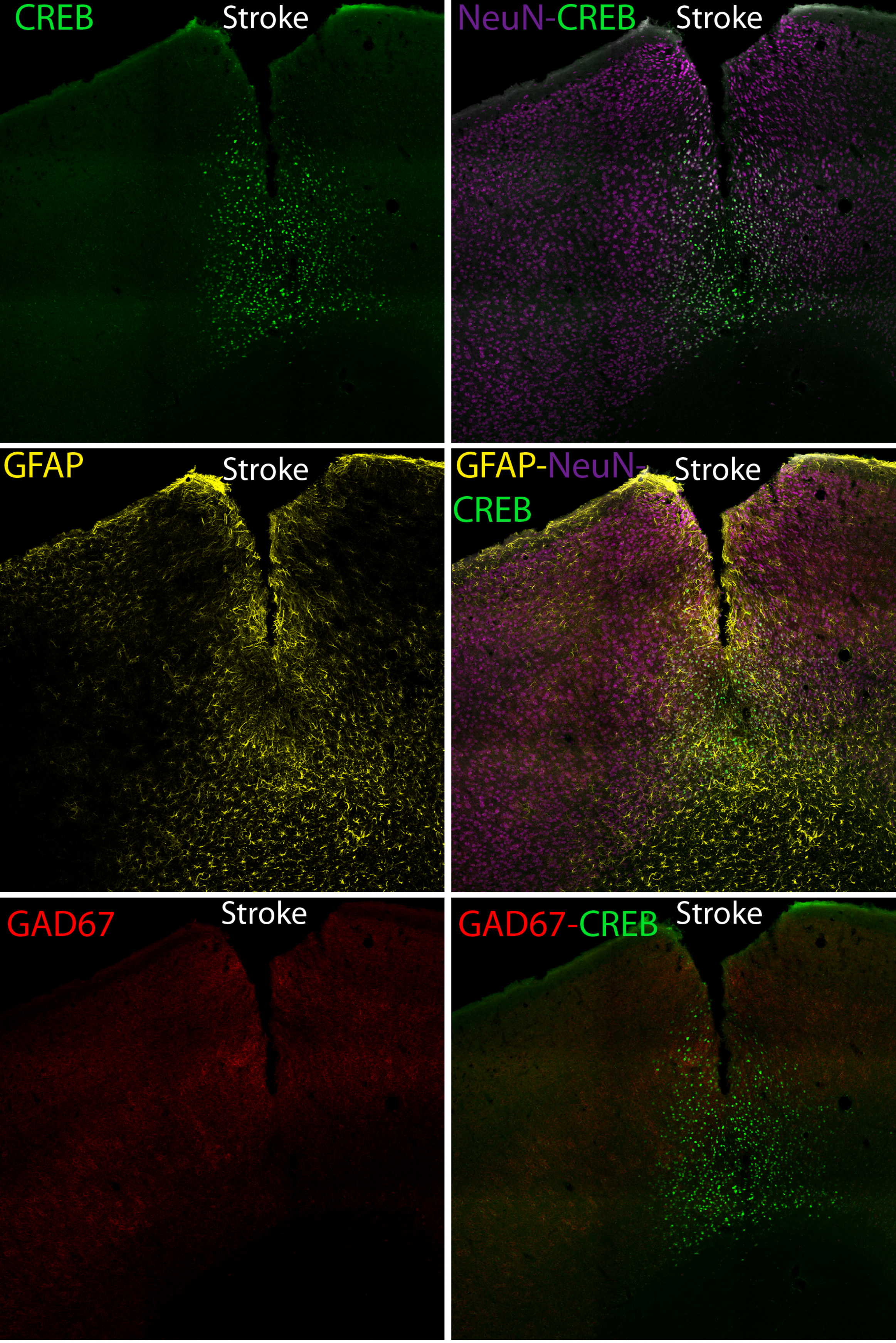
CREB
Stroke
NeuN-CREB
Stroke
GFAP
Stroke
GFAP-NeuN-
CREB
Stroke
GAD67
Stroke
GAD67-CREB
Stroke

Suppl Fig 4

Supplementary Figure 4. Lentiviral CREB transfection 1 week after stroke. Low magnification confocal images of same multi-fluorescent immunohistochemical staining as in Supplementary Figure 3. In each panel the top is the pial surface of cortex. The bottom is the subcortical white matter. These images are taken from coronal sections and anterior is to the left and ventral to the bottom. The stroke site is not visible but is to the right of the panels.

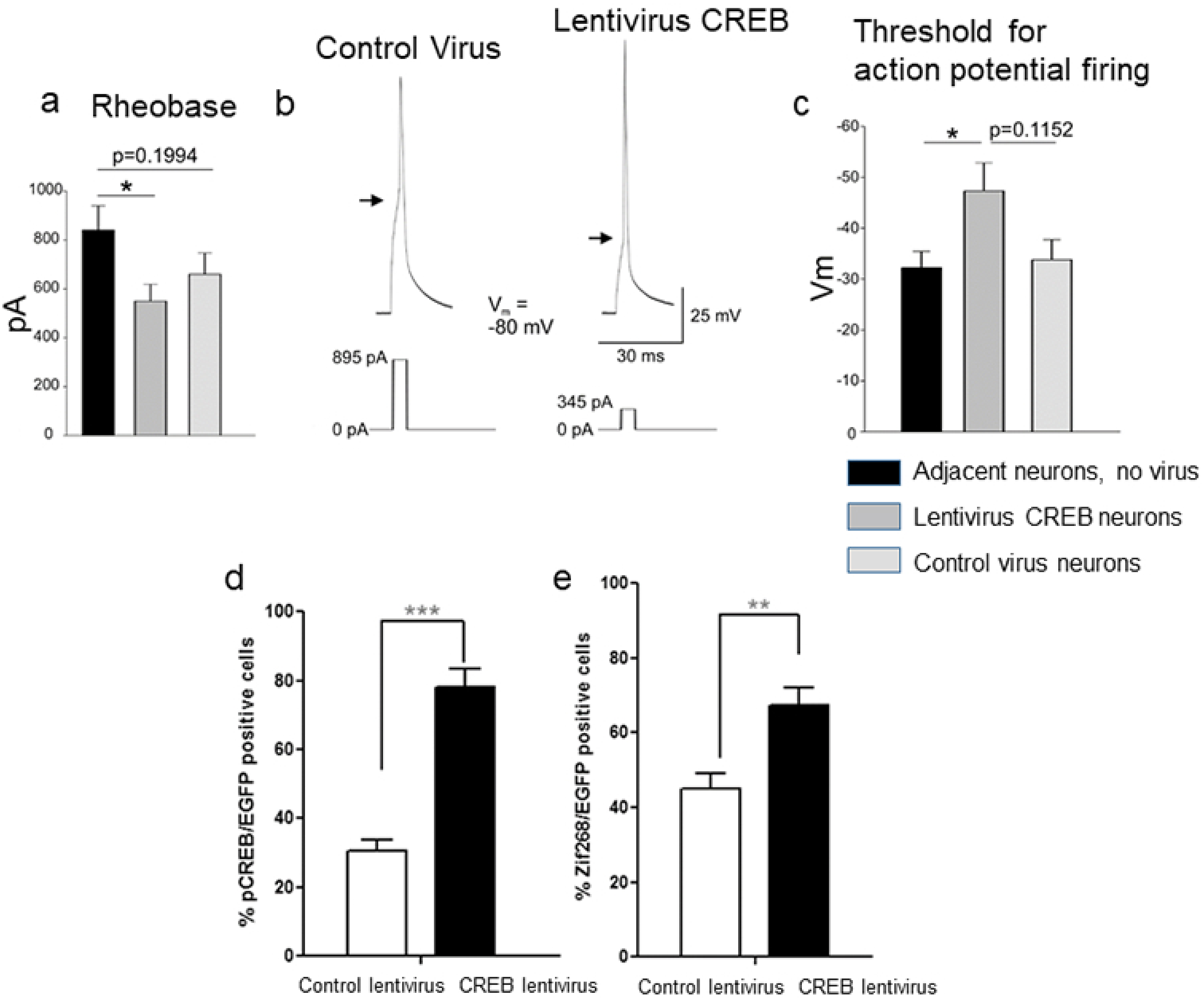
a
Rheobase
p=0.1994
*
pA
1000
800
600
400
200
0
b
Control Virus
Lentivirus CREB
Vm =
-80 mV
25 mV
30 ms
895 pA
0 pA
345 pA
0 pA
c
Threshold for
action potential firing
*
p=0.1152
Vm
-60
-50
-40
-30
-20
-10
0
Adjacent neurons, no virus
Lentivirus CREB neurons
Control virus neurons
d
***
% pCREB/EGFP positive cells
100
80
60
40
20
0
Control lentivirus
CREB lentivirus
e
**
% Zif268/EGFP positive cells
100
80
60
40
20
0
Control lentivirus
CREB lentivirus

Suppl Fig 5

Supplementary Figure 5. Increased excitability with lentivirus CREB transduction. (**a**-**c**). CREB expression leads to increased excitability in in cortical pyramidal neurons. CREB-tdTOM POS = lentivirus with CAMKIIa promoter CREB and tdTomato genes. tdTOM POS = control lentivirus, with CAMKIIA promoter and tdTomato. CREB-tdTOM NEG = non-fluorescent (virus negative) neurons adjacent to CREB transfected neurons in the same slice. (**a**) Rheobase measurements, with all cells held at a membrane potential of -80 mV. , Rheobase measurements were significantly smaller in CREB-containing cells (546.9+/-70.5 pA, n=8) when compared to non-CREB-containing cells (840.8+/-99.2 pA, n=6) in the same animal [t(12)=2.491, p=0.0284]. No differences were seen in rheobase measurements between adjacent cells no virus and control virus cells (660.0+/-88.5 pA, n=6)(t(10)=1.374, p=0.199) or between lentivirus CREB and control virus cells [t(12)=1.023, p=0.326]. (**b**). Raw traces of evoked action potentials in CPNs in response to a suprathreshold current pulse (5 ms). Arrows mark the start of the action potential. There is no difference in axon potential morphology between CREB-induced and non-induced neurons. (c) The membrane potential threshold for action potential (AP) firing was significantly lower in lenti CREB neurons (-47.7+/-18.1 mV, n=8) compared to control virus neurons (-30.2+/-3.0 mV, n=6) [t(12)=2.228, p=0.0458]. (**d**) Co-localization of pCREB with viral fluorescent protein in CREB lentivirus and control virus (t=14.82, df = 1, p=0.0429, two tailed T Test. (**e**) Colocalization of Zif268 with viral fluorescent protein in CREB lentivirus and control virus. Insert statistics (t=10.75, df=1, p=0.049, two tailed T test). All values are mean+/-SEM.

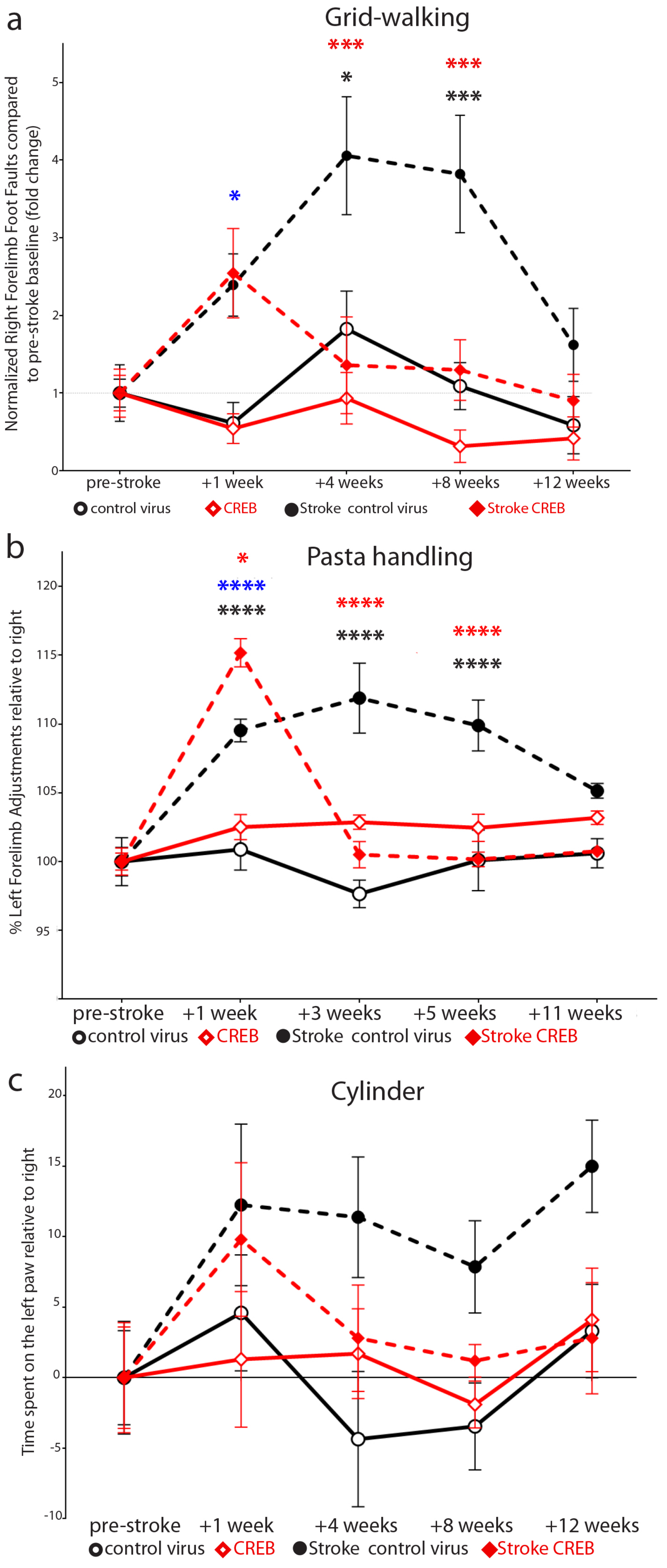

a

Stroke control virus vs. Stroke CREB
4 weeks: ***P < 0.001;
8 weeks: **P < 0.005

Stroke CREB vs. CREB
1 week: *P < 0.01

Stroke control virus vs. control virus
4 weeks: *P < 0.01;
8 weeks ***P < 0.001

ANOVA table	F (DFn, DFd)	P value
Interaction	F (12, 160) = 3.140	P = 0.0005
Row Factor	F (4, 160) = 5.170	P = 0.0006
Column Factor	F (3, 160) = 19.90	P < 0.0001

b

Stroke control virus vs. Stroke CREB
1 week: *P<0.01;
4 weeks: ****P < 0.0001;
8 weeks: ****P < 0.0001

Stroke CREB vs. CREB
1 week: *P < 0.01

Stroke control virus vs. control virus
4 weeks: ****P < 0.0001;
8 weeks: ****P < 0.0001

ANOVA table	F (DFn, DFd)	P value
Interaction	F (12, 160) = 11.13	P < 0.0001
Row Factor	F (4, 160) = 19.14	P < 0.0001
Column Factor	F (3, 160) = 36.26	P < 0.0001

c

ANOVA table	F (DFn, DFd)	P value
Interaction	F (12, 170) = 0.659	P = 0.7883
Row Factor	F (4, 170) = 2.636	P = 0.0358
Column Factor	F (3, 170) = 5.799	P = 0.0008

Suppl Fig 6

Supplementary Figure 6. Behavioral results from lentiviral CREB transfection in motor cortex after stroke. These panels are the same as in Fig. 2b but with all statistical testing reported on the images and the inclusion of the cylinder testing studies. Values are mean+/-SEM.

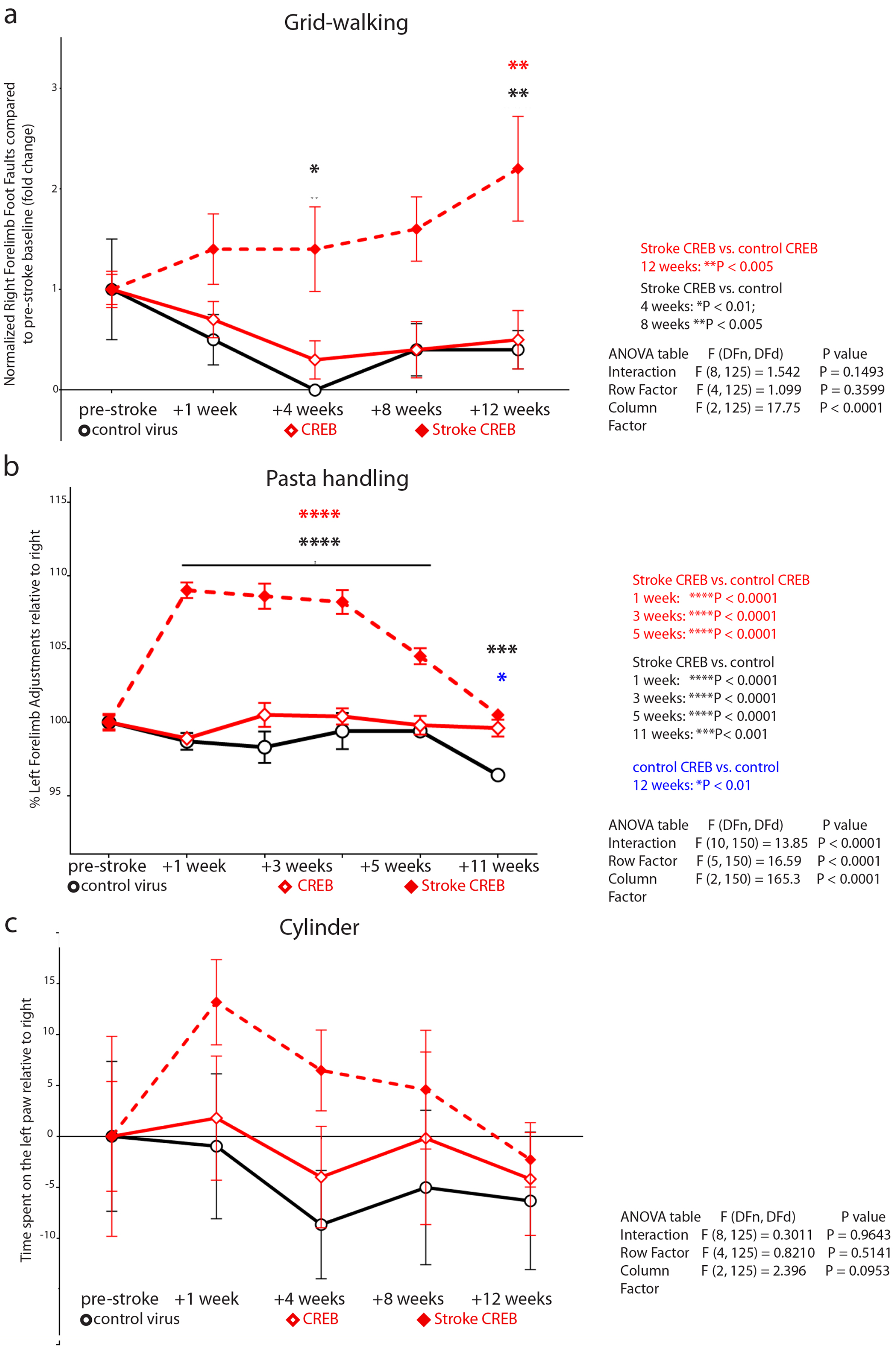

a
Grid-walking
Normalized Right Forelimb Foot Faults compared to pre-stroke baseline (fold change)
0
1
2
3
**
**
*
pre-stroke +1 week +4 weeks +8 weeks +12 weeks
control virus
CREB
Stroke CREB
Stroke CREB vs. control CREB
12 weeks: **P < 0.005
Stroke CREB vs. control
4 weeks: *P < 0.01;
8 weeks **P < 0.005
ANOVA table F (DFn, DFd) P value
Interaction F (8, 125) = 1.542 P = 0.1493
Row Factor F (4, 125) = 1.099 P = 0.3599
Column Factor F (2, 125) = 17.75 P < 0.0001
b
Pasta handling
% Left Forelimb Adjustments relative to right
95
100
105
110
115
****
****
***
*
pre-stroke +1 week +3 weeks +5 weeks +11 weeks
control virus
CREB
Stroke CREB
Stroke CREB vs. control CREB
1 week: ****P < 0.0001
3 weeks: ****P < 0.0001
5 weeks: ****P < 0.0001
Stroke CREB vs. control
1 week: ****P < 0.0001
3 weeks: ****P < 0.0001
5 weeks: ****P < 0.0001
11 weeks: ***P< 0.001
control CREB vs. control
12 weeks: *P < 0.01
ANOVA table F (DFn, DFd) P value
Interaction F (10, 150) = 13.85 P < 0.0001
Row Factor F (5, 150) = 16.59 P < 0.0001
Column Factor F (2, 150) = 165.3 P < 0.0001
c
Cylinder
Time spent on the left paw relative to right
15
10
5
0
-5
-10
pre-stroke +1 week +4 weeks +8 weeks +12 weeks
control virus
CREB
Stroke CREB
ANOVA table F (DFn, DFd) P value
Interaction F (8, 125) = 0.3011 P = 0.9643
Row Factor F (4, 125) = 0.8210 P = 0.5141
Column Factor F (2, 125) = 2.396 P = 0.0953

Suppl Fig 7

Supplementary Figure 7. Behavioral results from lentiviral CREB transfection in parietal association cortex after stroke. These panels are the same as in Fig. 2d,e but with all statistical testing reported on the images and the inclusion of the cylinder testing studies. Values are mean+/-SEM.

a

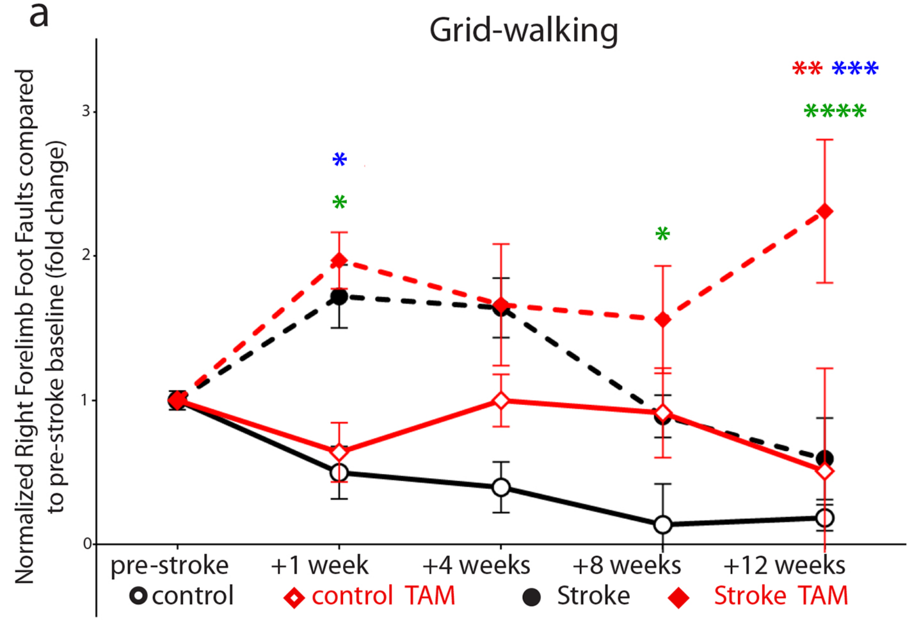

Stroke TAM vs. Stroke
12 weeks: **P < 0.005;

Stroke TAM vs. TAM
1 week: *P < 0.01
12 week: ***P < 0.001

Stroke vs. control
4 weeks: *P < 0.01;
8 weeks ***P < 0.001

Stroke TAM vs. control
1 week: *P < 0.01
8 weeks: *P < 0.01
12 week: ****P < 0.0001

ANOVA table	F (DFn, DFd)	P value
Interaction	F (12, 180) = 2.269	P = 0.0106
Row Factor	F (4, 180) = 1.153	P = 0.3332
Column Factor	F (3, 180) = 17.57	P < 0.0001

b

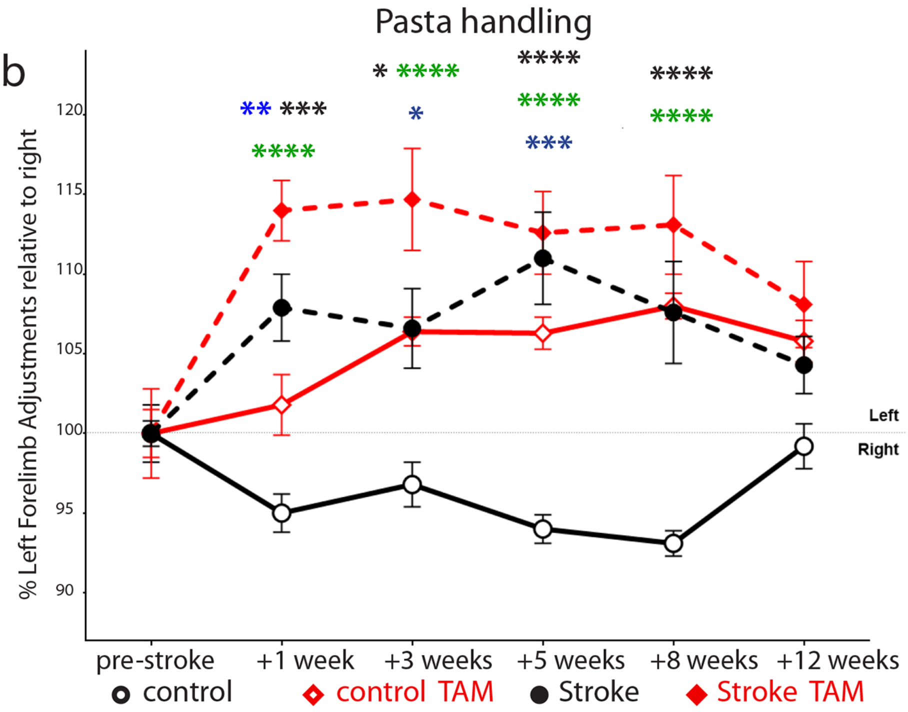

Stroke TAM vs. TAM
1 week: **P < 0.005

Stroke vs. control
1 week: ***P < 0.001
3 weeks: *P < 0.01;
5 weeks ****P < 0.0001
7 weeks ****P < 0.0001

Stroke TAM vs. control
1 week: ****P < 0.0001
3 weeks: ****P < 0.0001
5 weeks: ****P < 0.0001
7 weeks: ****P < 0.0001

TAM vs. control
3 weeks: *P < 0.01
5 weeks: ***P < 0.005
7 weeks: ****P < 0.0001

ANOVA table	F (DFn, DFd)	P value
Interaction	F (15, 204) = 3.448	P < 0.0001
Row Factor	F (5, 204) = 5.102	P = 0.0002
Column Factor	F (3, 204) = 51.27	P < 0.0001

c

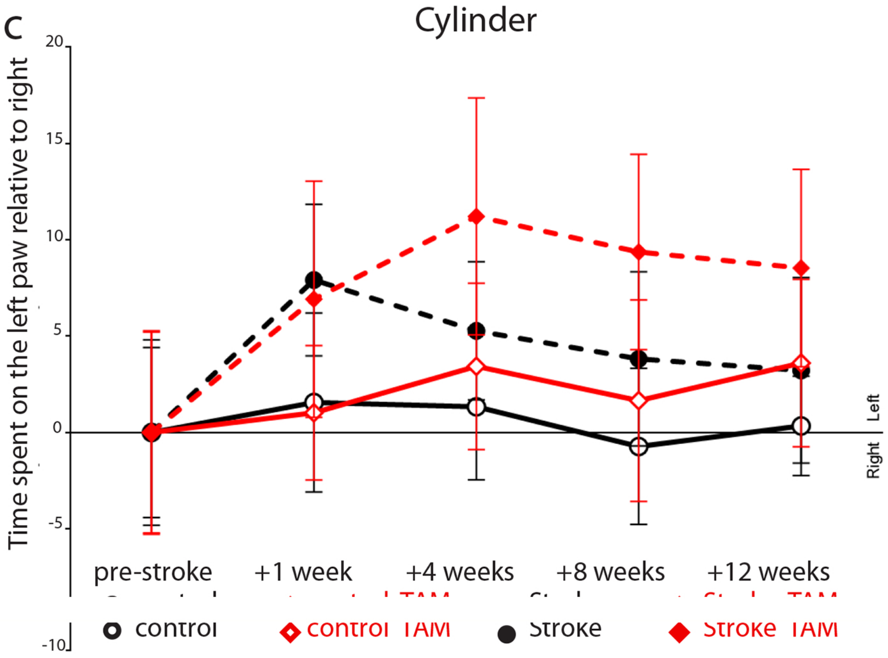

ANOVA table	F (DFn, DFd)	P value
Interaction	F (12, 180) = 0.2143	P = 0.9977
Row Factor	F (4, 180) = 0.7598	P = 0.5527
Column Factor	F (3, 204) = 51.27	P < 0.0001

Suppl Fig 8

Supplementary Figure 8. Behavioral results from lentiviral CREB transfection in parietal association cortex after stroke, area PTLp. These panels are the same as in Fig. 2f,g but with all statistical testing reported on the images and the inclusion of the cylinder testing studies. Values are mean+/-SEM.

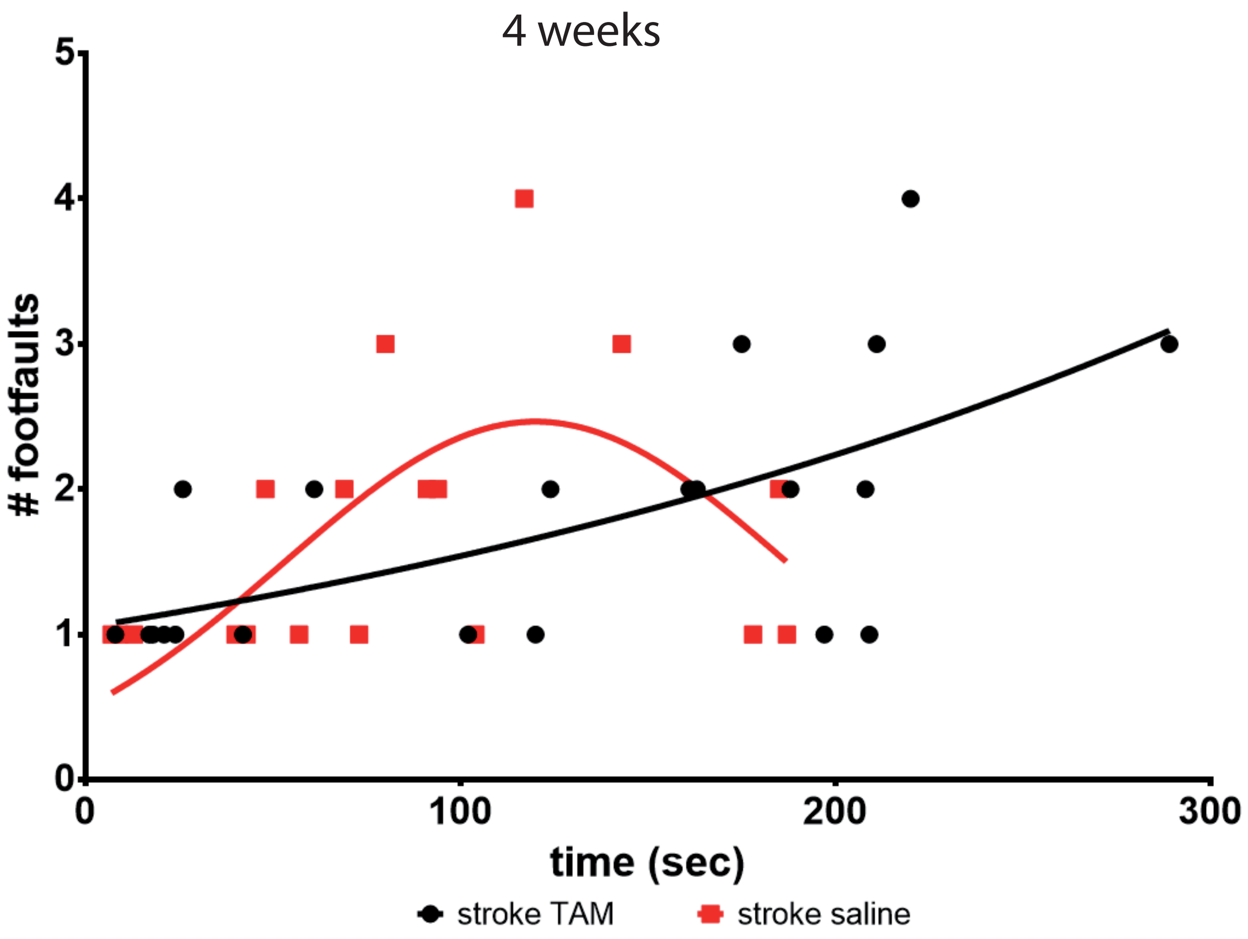
4 weeks
footfaults
0
1
2
3
4
5
0
100
200
300
time (sec)
stroke TAM
stroke saline

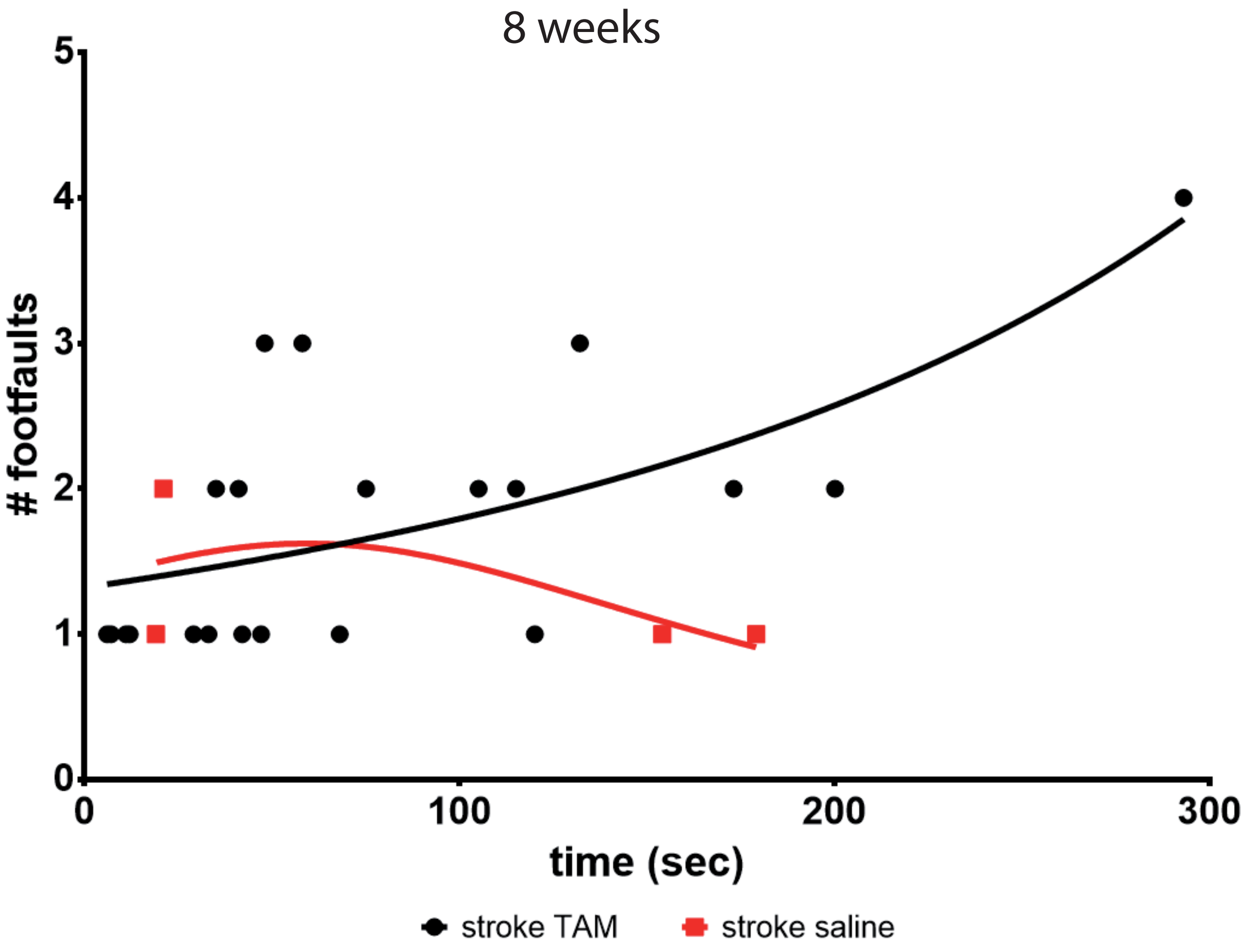
8 weeks
footfaults
0
1
2
3
4
5
0
100
200
300
time (sec)
stroke TAM
stroke saline

Suppl Fig 9

Supplementary Figure 9. Analysis of footfaults over time after stroke. The number of footfaults (y axis) is plotted over time in the testing session 4 weeks after stroke (**a**) and 8 weeks after stroke (**b**). The x axis indicates time after placement of the mouse on the grid. Most gridwalking errors in stroke without CREB blockade occur in the early parts of the testing session. With CREB blockade footfaults are spread over the course of the testing session. All of the data for each testing session is presented in Fig. 2f.

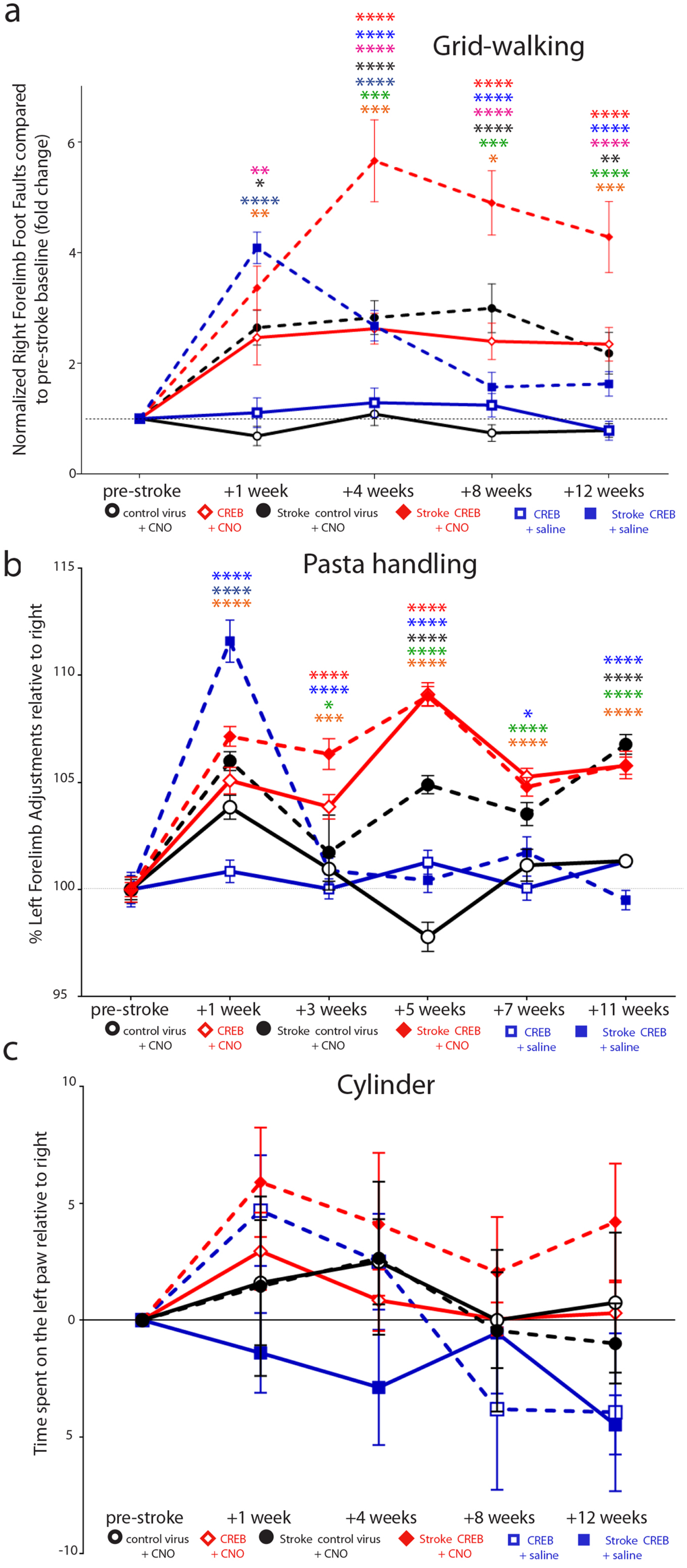

Stroke control virus CNO vs. Stroke CREB CNO (*)

Stroke CREB saline vs. Stroke CREB CNO (*)

Stroke CREB CNO vs. CREB CNO (*)

Stroke control virus CNO vs. control virus CNO (*)

Stroke CREB saline vs. CREB saline (*)

Control virus CNO vs. CREB CNO (*)

CREB saline vs. CREB CNO (*)

*P < 0.01, **P < 0.005,
***P < 0.001, ****P < 0.0001

ANOVA table	F (DFn, DFd)	P value
Interaction	F (20, 300) = 12.44	P < 0.0001
Row Factor	F (4, 300) = 49.68	P < 0.0001
Column Factor	F (5, 300) = 98.15	P < 0.0001

Stroke control virus CNO vs. Stroke CREB CNO (*)

Stroke CREB saline vs. Stroke CREB CNO (*)

Stroke CREB CNO vs. CREB CNO (ns)

Stroke control virus CNO vs. control virus CNO (*)

Stroke CREB saline vs. CREB saline (*)

Control virus CNO vs. CREB CNO (*)

CREB saline vs. CREB CNO (*)

*P < 0.01, **P < 0.005,
***P < 0.001, ****P < 0.0001

ANOVA table	F (DFn, DFd)	P value
Interaction	F (25, 372) = 14.33	P < 0.0001
Row Factor	F (5, 372) = 53.62	P < 0.0001
Column Factor	F (5, 372) = 63.86	P < 0.0001

ANOVA table	F (DFn, DFd)	P value
Interaction	F (20, 285) = 0.6644	P = 0.8599
Row Factor	F (4, 285) = 2.451	P = 0.0463
Column Factor	F (5, 285) = 2.849	P = 0.0158

Suppl Fig 10

Supplementary Figure 10. Behavioral results of inhibitory DREADD activation in control and CREB-transfected neurons after stroke. The panels are the same as in Fig. 3 but present all of the statistical comparisons and the cylinder testing studies. Values are mean+/-SEM.

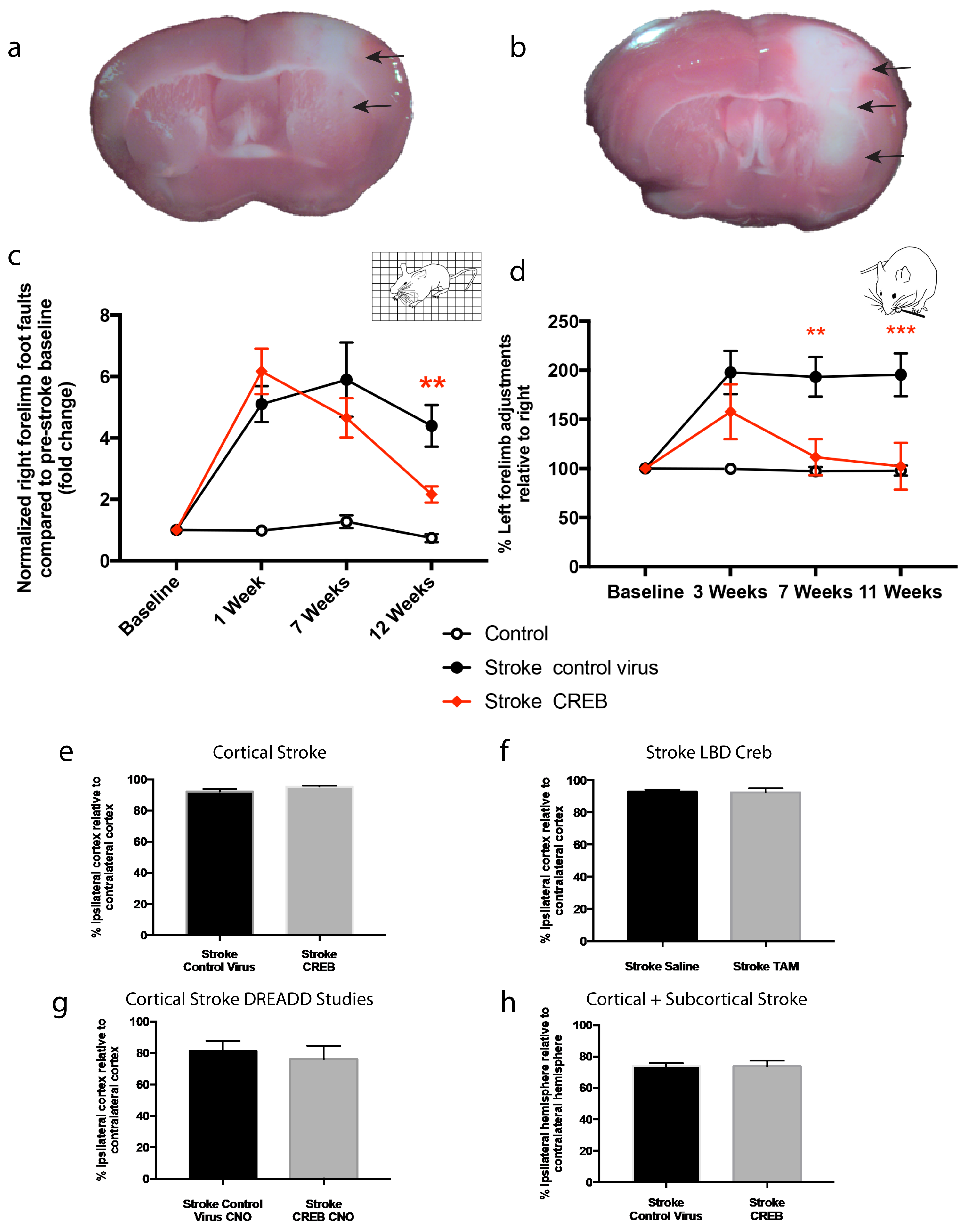
a
b
c
Normalized right forelimb foot faults compared to pre-stroke baseline (fold change)
8
6
4
2
0
**
Baseline
1 Week
7 Weeks
12 Weeks
d
% Left forelimb adjustments relative to right
250
200
150
100
50
0
**
***
Baseline
3 Weeks
7 Weeks
11 Weeks
Control
Stroke control virus
Stroke CREB
e
Cortical Stroke
% Ipsilateral cortex relative to contralateral cortex
100
80
60
40
20
0
Stroke Control Virus
Stroke CREB
f
Stroke LBD Creb
% Ipsilateral cortex relative to contralateral cortex
100
80
60
40
20
0
Stroke Saline
Stroke TAM
g
Cortical Stroke DREADD Studies
% Ipsilateral cortex relative to contralateral cortex
100
80
60
40
20
0
Stroke Control Virus CNO
Stroke CREB CNO
h
Cortical + Subcortical Stroke
% Ipsilateral hemisphere relative to contralateral hemisphere
100
80
60
40
20
0
Stroke Control Virus
Stroke CREB

Suppl Fig 11

Supplementary Figure 11. Cortical and Subcortical (Large) Stroke Model and Measurement of Stroke Sizes across Studies. (**a**, **b**) TTC staining of cortical and striatal stroke at three days after the infarct. The pale area, highlighted by arrows, is the infarct. This is located in the motor cortex, subcortical white matter and striatum. (**c**) Gridwalking task of forelimb function in gait in cortical and striatal stroke model. Y axis is percentage of footfaults of the right (affected) forelimb contralateral to the stroke. (**d**) Pasta handling task of distal forelimb function in cortical and striatal stroke. Y axis is the percentage of left forelimb adjustments (unaffected forepaw) relative to right forepaw (affected forepaw). Lentivirus CREB gain of in motor cortex produced a significant recovery in forelimb function compared with stroke + Control virus (***$p < 0.005$) at 12 weeks in gridwalking and 7 and 11 weeks in pasta handling (insert p values). (**e**) Measurement of stroke sizes for studies in Fig. 2b,c. This measures the size difference in cortex on the side of the stroke to that of the contralateral (non-stroke) side $p = 0.1042$, $t=1.913$ df=6. The normal (non-stroke) value is 100%: the two cortex on the two hemispheres are the same size. (**f**). Stroke size measurement in LBD-Creb mice for studies in Fig. 2f,g. $p = 0.9076$, $t=0.1211$ df=6. (**g**) Stroke size measurement in cortical stroke DREADD studies in Fig. 3d-h. $p = 0.6391$, $t=0.4937$ df=6. (**h**) Stroke size in cortical and striatal stroke in Supplementary Figure 11c,d. For measurements in this stroke model, the size of the entire ipsilateral hemisphere was divided by the contralateral hemisphere. $p = 0.9720$, $t=0.03664$ df=6. Values are mean+/-SEM.

a

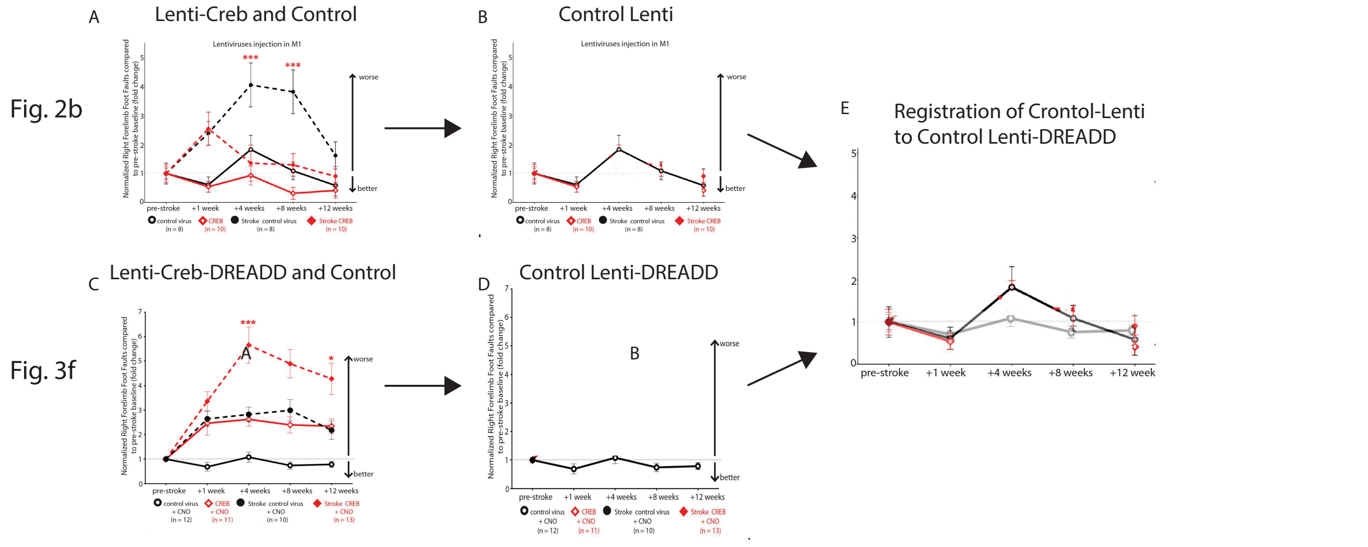

b

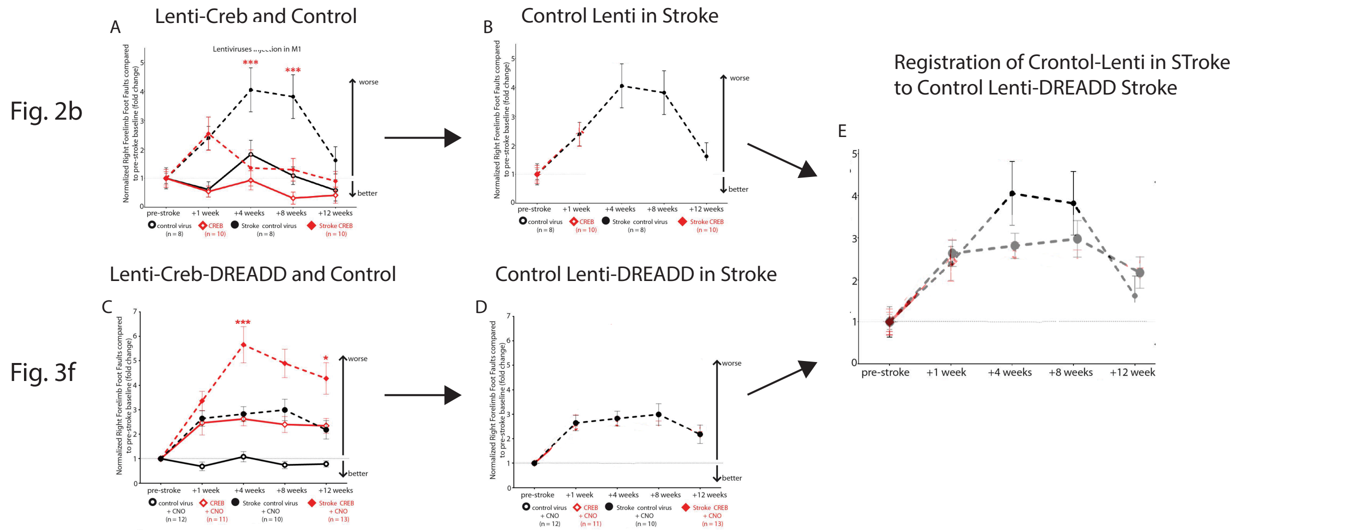

c

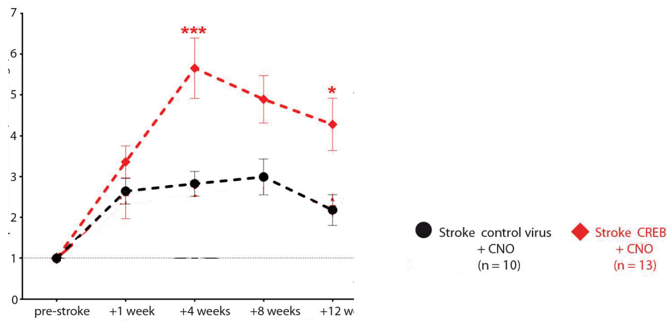

Suppl Fig 12

Supplementary Figure 12. (**a**) Effect of effect of inhibitory DREADD in motor cortex in control (non-stroke) mice on gait. The data from the two behavioral studies, lentivirus control and lentivirus-inhibitory DREADD control, were separately compared to determine if the presence of inhibitory DREADD activation by itself impairs motor control. The data on gridwalking from Fig. 2b and Fig. 3f was isolated to look at motor performance in the conditions of control lentivirus and lentivirus-DREADD+CNO (i.e. with the inhibitory DREADD effect). There is some variance in motor performance over the testing periods, but no significant difference between the two groups ($f(1,90)=1.27$, $p=0.2634$). (**b**) Effect of inhibitory DREADD in motor cortex in stroke mice on gait. The same data as in (a) were separately compared for stroke+control lentivirus vs. stroke+lentivirus-DREADD+CNO. Both groups have worsening motor control after stroke, as seen in the increase in footfaults. There is a non-significant difference between control-lenti in stroke and control-lenti-DREADD+CNO in stroke at 4 weeks, but these two groups are otherwise overlapping in their behavioral performance ($f(1,80) = 1.44$, $p=0.2293$). (**c**). Compare the effect of activation of the inhibitory DREADD in control (a) and stroke (b) to the effect of activation of the inhibitory DREADD after CREB induction in stroke. Note that the scale is higher for Y in this graph than for (a,b), because the effect on motor control is much greater after after CREB induction than the non-significant effect of just inhibitory DREADD induction without CREB.

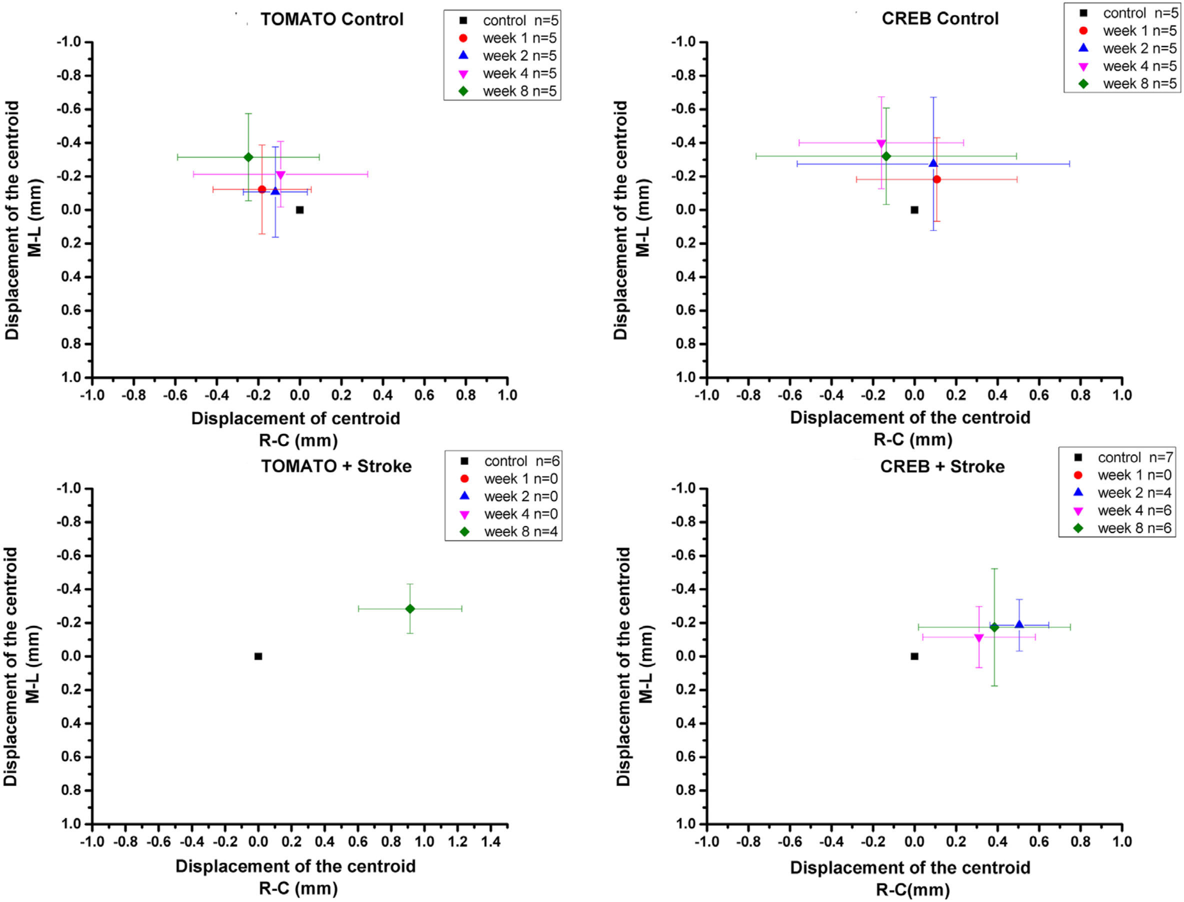
TOMATO Control
control n=5
week 1 n=5
week 2 n=5
week 4 n=5
week 8 n=5
Displacement of the centroid M-L (mm)
Displacement of centroid R-C (mm)
CREB Control
control n=5
week 1 n=5
week 2 n=5
week 4 n=5
week 8 n=5
Displacement of the centroid M-L (mm)
Displacement of the centroid R-C (mm)
TOMATO + Stroke
control n=6
week 1 n=0
week 2 n=0
week 4 n=0
week 8 n=4
Displacement of the centroid M-L (mm)
Displacement of the centroid R-C (mm)
CREB + Stroke
control n=7
week 1 n=0
week 2 n=4
week 4 n=6
week 8 n=6
Displacement of the centroid M-L (mm)
Displacement of the centroid R-C(mm)

Suppl Fig 13

Supplementary Figure 13. Remapping of forepaw somatosensory cortex after stroke. Top row shows location of center of forepaw S1 cortex in lentivirus control with the fluorescent reporter tdTomato, and with CREB. Over time there is no significant shift in the location of the S1 forepaw location. Middle row shows the location of the forepaw S1 cortex center after stroke. In TOMATO+STROKE (middle row, left panel) there is no activation in cortex from forepaw stimulation in weeks 1, 2, and 4 (second row of images from top in Fig. 5c). In stroke with control (non-CREB) induction there is a significant long distance shift of the center of the forepaw location. In CREB induction after stroke there is non-significant shift in location. The statistical testing of this data is in Fig. 5d.

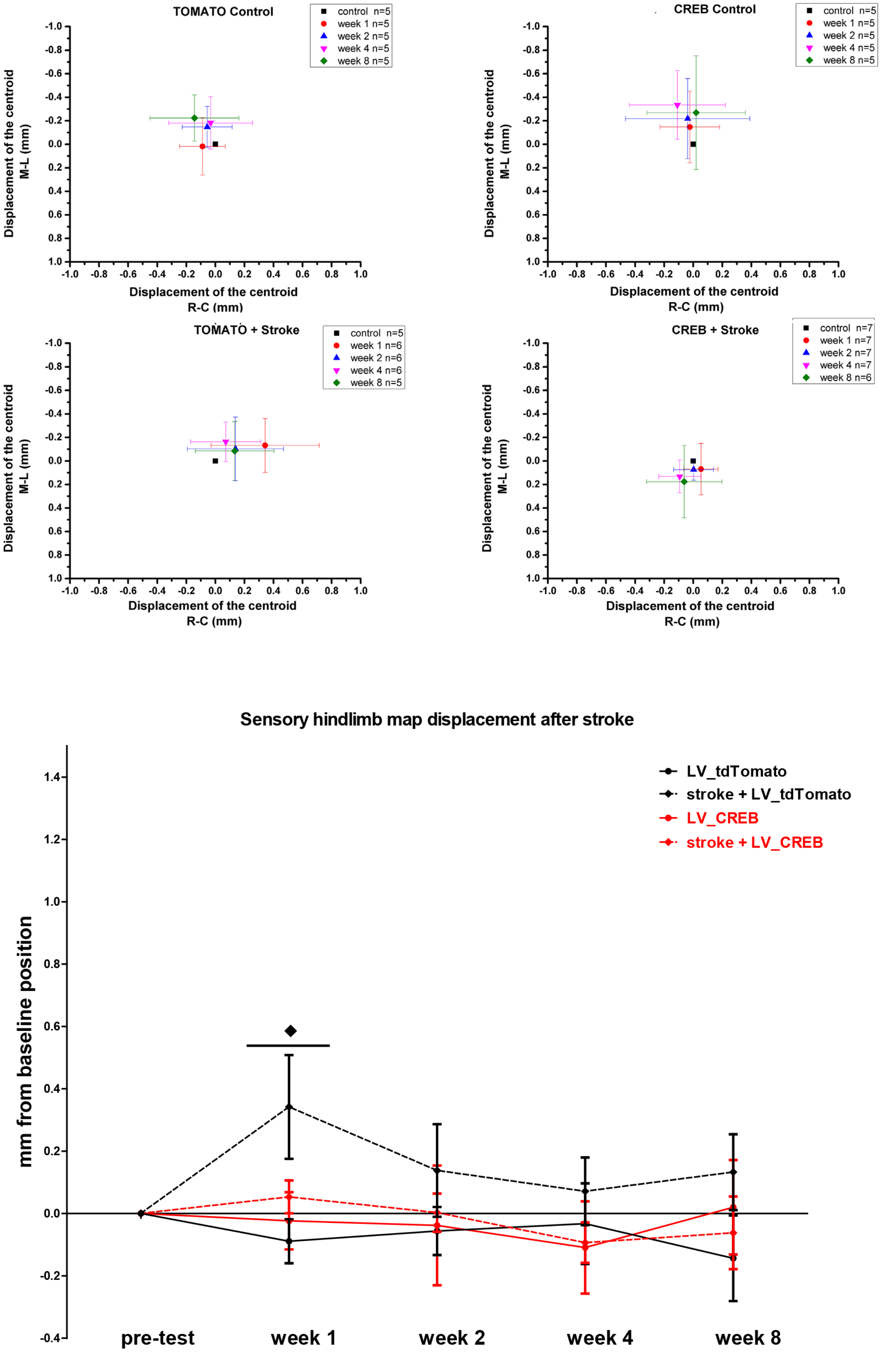

TOMATO Control
control n=5
week 1 n=5
week 2 n=5
week 4 n=5
week 8 n=5
CREB Control
control n=5
week 1 n=5
week 2 n=5
week 4 n=5
week 8 n=5
TOMATO + Stroke
control n=5
week 1 n=6
week 2 n=6
week 4 n=6
week 8 n=5
CREB + Stroke
control n=7
week 1 n=7
week 2 n=7
week 4 n=7
week 8 n=6
Displacement of the centroid M-L (mm)
Displacement of the centroid R-C (mm)
-1.0
-0.8
-0.6
-0.4
-0.2
0.0
0.2
0.4
0.6
0.8
1.0
Sensory hindlimb map displacement after stroke
LV_tdTomato
stroke + LV_tdTomato
LV_CREB
stroke + LV_CREB
mm from baseline position
1.4
1.2
1.0
0.8
0.6
0.4
0.2
0.0
-0.2
-0.4
pre-test
week 1
week 2
week 4
week 8

Suppl Fig 14

Supplementary Figure 14. Hindpaw somatosensory cortex does not remap after stroke. Same conventions as in Supplementary Figure 11. Compare the bottom panel with Fig. 5d. There is an early shift of the hindpaw somatosensory cortex at week 1 that is not sustained.

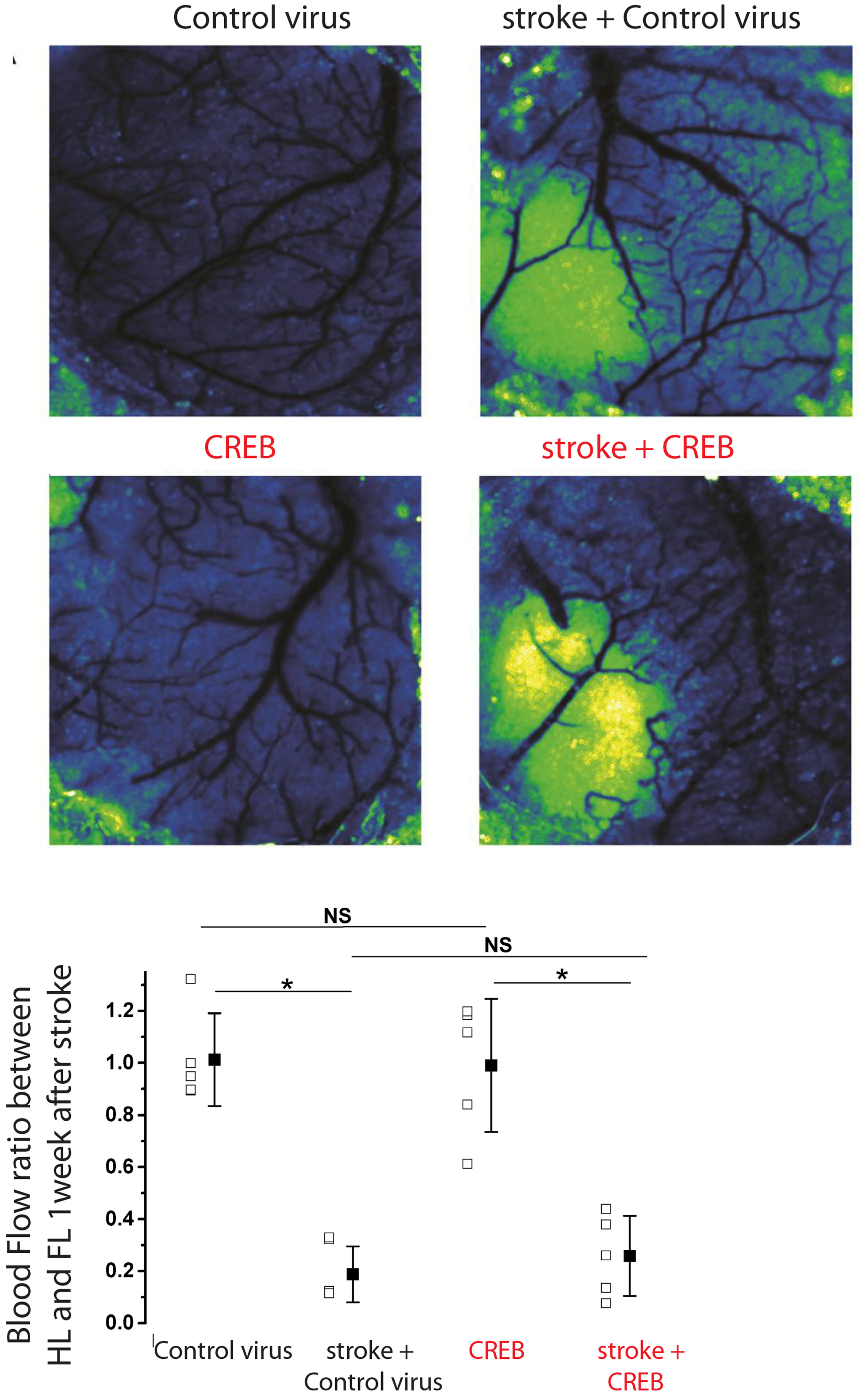
Control virus
stroke + Control virus
CREB
stroke + CREB
NS
NS
*
*
Blood Flow ratio between HL and FL 1week after stroke
1.2
1.0
0.8
0.6
0.4
0.2
0.0
Control virus
stroke + Control virus
CREB
stroke + CREB

Suppl Fig 15

Supplementary Figure 15. Laser speckle contrast imaging of cerebral blood flow 1 week after stroke: Laser speckle contrast imaging was performed through the cranial window at different intervals before and after stroke. The cortical surface was illuminated with an expanded laser diode beam (785 nm, 80mW) coupled to a 600 μm diameter fiber optic cable. Blue color represent regular blood flow while green-yellow show the reduced blood flow in the stroke area. Top row shows laser speckle imaging of control (left) and stroke control virus (right) one week after stroke. Center row shows laser speckle imaging of CREB alone (left) and stroke CREB (right) one week after stroke. Bottom row show the quantification of the cortical blood flow between stroke and relative control. Relative cortical blood flow values were obtained as the ratio K_{02}/K_{t2}.
