# Supplementary material for: CREB controls cortical circuit plasticity and functional recovery after stroke
Source: Nat Commun. 2018 Jun 8;9:2250. doi: 10.1038/s41467-018-04445-9 (PMC5993731; doi:10.1038/s41467-018-04445-9)
Supplement: Supplementary file 2 — Peer Review File [file 41467_2018_4445_MOESM2_ESM.pdf]

Editorial Note: this manuscript has been previously reviewed at another journal that is not operating a transparent peer review scheme. This document only contains reviewer comments and rebuttal letters for versions considered at Nature Communications.

REVIEWERS' COMMENTS:

Reviewer #1 (Remarks to the Author):

The authors have addressed the comments of the referees satisfactorily. The model of large stroke used is complex and involves pharmacological manipulations. But this model is the only one available to the authors and, in the final analysis, does indicate protection also in a model with more extensive tissue injury.

Reviewer #2 (Remarks to the Author):

The authors have done a good job of addressing my concerns. I feel the paper will add significant new knowledge for the stroke field. While there are some issues with the effects of CREB in non-stroke animals (raised by reviewers), the authors explanations and logic for why the plastic effects are significant for recovery were well taken.
